# Prevalence and incidence of post-traumatic stress disorder and symptoms in people with chronic somatic diseases: A systematic review and meta-analysis

**DOI:** 10.3389/fpsyt.2023.1107144

**Published:** 2023-01-18

**Authors:** Frederike Lunkenheimer, Patricia Garatva, Lena Steubl, Harald Baumeister

**Affiliations:** Department of Clinical Psychology and Psychotherapy, Institute of Psychology and Education, Ulm University, Ulm, Germany

**Keywords:** post-traumatic stress disorder (PTSD), post-traumatic stress symptoms, prevalence, incidence, systematic review, meta-analysis, chronic somatic illness

## Abstract

**Introduction:**

Comprehensive evidence on prevalence and incidence of post-traumatic stress disorder (PTSD) and symptoms (PTSS) in people with chronic somatic diseases (CD) is lacking.

**Objective:**

To systematically and meta-analytically examine prevalence and incidence of PTSD and PTSS in people with CD compared with people without CD.

**Methods:**

MEDLINE, Embase, and PsycINFO were searched from inception (1946) to June 2020. Studies reporting point, 12-month, lifetime prevalence, or 12-month incidence of PTSD and PTSS in people with CD were selected and reviewed in accordance with PRISMA guidelines by two independent reviewers. Risk of bias was assessed by a combination of the Newcastle-Ottawa Scale and recommendations of the Cochrane Collaboration for non-comparative studies. Pooled estimates were calculated using random effects meta-analyses. Between-study heterogeneity was assessed using the I2 statistic.

**Results:**

Data were extracted from studies reporting on point prevalence (*k* = 60; *n* = 21,213), 12-month prevalence (*k* = 3; *n* = 913), and lifetime prevalence (*k* = 6; *n* = 826). 12-month incidence estimates were not available. The pooled estimate for the point prevalence of PTSD (*k* = 41) across CD was 12.7% (95% CI, 8.6 to 18.4%) and 19.6% regarding PTSS (13.2 to 28.1%; *k* = 24). Individuals with cerebrovascular disorder (*k* = 4) showed the highest pooled point prevalence for PTSD (23.6%, 95% CI, 16.8 to 32.0%), those with cardiovascular diseases the lowest (6.6%, 1.9 to 20.9%; *k* = 5). The pooled 12-month prevalence of PTSD (*k* = 3) was 8.8% (95% CI, 5.5 to 13.5%) and the lifetime prevalence (*k* = 6) was 12.1% (7.6 to 18.5%). Pooled estimates of PTSD prevalence in people with compared to those without CD showed an odds ratio of 9.96 (95% CI, 2.55 to 38.94; *k* = 5).

**Conclusion:**

Post-traumatic stress disorder and PTSS are common and substantially higher in people with compared to those without CD. Earlier detection and treatment of this comorbidity might improve mental and physical health, reduce the incidence of further diseases, and reduce mortality.

**Clinical trial registration:**

https://osf.io/9xvgz, identifier 9xvgz.

## Introduction

Chronic somatic diseases (CD) such as cardiovascular diseases, cancers, respiratory diseases, and diabetes account for 71% of all deaths worldwide; this is equivalent to 41 million deaths per year (1). More than 40% of people with CD also have a mental disorder; this is twice the 12-month prevalence of people without CD (2). Most studies refer to anxiety, depression, and somatoform disorders (2), while knowledge on prevalence and incidence of other mental disorders such as post-traumatic stress disorder (PTSD) is limited. Hereby, PTSD refers to a trauma- and stressor-related disorder, caused by a traumatic event, such as threatened death, serious injury [including a life-threatening condition such as CD (3)], or sexual violence (4).

From diagnostic and statistical manual of mental disorders (DSM)-4 to DSM-5 the requirements for the fulfilment of the traumatic event were restricted. A threat to physical integrity - and thus a CD diagnosis - is no longer considered a criterion for a traumatic event that serves as the basis for a PTSD diagnosis (3, 4) unless CD is associated with increased mortality (4). Increased mortality has been demonstrated in CD such as neurological conditions (5), musculoskeletal disorders (6), heart disease, cerebrovascular disease and cancer (7), among others. Thus, CD, which may be experienced as an aversive event given the experience of diagnosis and medical treatment, may be a possible trigger for elevated post-traumatic stress symptoms (PTSS) and, with increased mortality, may also be a possible trigger for PTSD (4). On the other hand, it has already been shown, that PTSD can be a risk factor for developing CD (8–10), and a negative prognostic factor regarding disease outcomes (11–13) and treatment adherence (12, 14). Even PTSS show a significant negative impact on CD severity, treatment adherence, health problems, and functional impairment (15–18). People with PTSD show metabolic dysfunction, alterations in inflammatory pathways, and neuroendocrine dysfunction that have not been demonstrated in people without PTSD (8, 19, 20). The pathophysiology is associated with CD, the incidence of further CD (such as cardiovascular disease and/or diabetes), poorer health recovery, and worse treatment outcomes (10).

Prevalences of PTSD or PTSS in people with CD can provide insights into the frequency and relevance of this comorbidity. Studies on prevalences of PTSD usually refer to specific diseases such as chronic pain with a pooled mean prevalence of 9.7% (95% CI, 5.2 to 17.1) (21) or cardiovascular disease with an average prevalence of 12% (0 to 38%) (22). Overall, there exist systematic reviews for only a few diseases (e.g., chronic pain, cancer, acute coronary syndrome, cardiovascular disease) (21–24) with different prevalences depending on the type of specific disease [e.g., cancer type or chronic pain type (21, 25)]. The systematic reviews find higher prevalences for the assessment with self-reporting questionnaires than with structured interviews (21, 24, 25), and show inconsistent results regarding the effects of moderators time of PTSD assessment since CD diagnosis or initial treatment, setting, age, and gender (21, 23, 25) on prevalences. Preliminary findings also suggest a substantially increased risk for PTSD in people with CD compared to those without CD. For example, organ transplant recipients are shown to be two to five times more likely to have comorbid PTSD than the general population (26). Studies that report the incidence of PTSD or PTSS following a CD event may provide insight into the impact of a CD event on PTSD or PTSS.

The discrepant estimates for specific CD limit comparisons between different diseases. Integrated information about the prevalence and incidence of PTSD or PTSS in people with CD (across CDs) is lacking, which is essential for an overall insight into the relevance of the topic. Therefore, the present systematic review and meta-analysis aimed to examine:

1. What are the prevalence rates (point, 12-month, lifetime) of PTSD or PTSS?
2. Is there an increased risk for comorbid PTSD or PTSS in people with compared to without CD?
3. What are the 12-month incidence rates of PTSD or PTSS?

Each (1.-3.) (a) pooled across CDs, and

(b) examined for association with possible related variables (e.g., specific CD categories, assessment method, time of PTSD assessment since CD diagnosis or initial treatment, duration of CD, age, setting, gender, ethnicity, study quality, and publication year).

## Methods

### Study design and registration

We conducted a systematic review and meta-analysis in accordance with Preferred Reporting Items for Systematic Reviews and Meta-Analyses (PRISMA) guidelines (27). The predefined protocol was registered at Open Science Framework (OSF; identification number 9xvgz; date of registration: February 28th, 2020).

### Inclusion criteria and outcomes

We predefined the following inclusion criteria: (1) original studies without restriction regarding publication status (i.e., peer-reviewed full-text journal articles, non-peer-reviewed full-text manuscripts, and conference abstracts) to avoid data limitation, (2) reporting on or including data allowing the calculation of: point (≤4 weeks), 12-month, lifetime prevalence, or 12-month incidence of PTSD [i.e., diagnosed PTSD through structured interviews or the assessment based on PTSD according to DSM-5 (4) or ICD-10 (28) or prior DSM/ICD versions] and/or PTSS (i.e., assessment providing an indication for elevated PTSS but is not based on classification system) in people with (3) CD [defined following the definition of Kampling et al. (29) who specified a list of ICD-10 diagnoses to meet the criteria of CD]. Studies had to be available in (4) English or German language. If no full text was available even after contacting the authors, this led to exclusion.

### Literature search

The literature search (including backward searches) was performed using the electronic databases MEDLINE, Embase, and PsycINFO from 13.03.2020 to 24.07.2020. Publications from their inception to June 2020 were considered. The search string combines terms related to CD/CD events with those related to PTSD/PTSS. Proximity operators were used and MeSH (Medical Subject Headings) terms were applied where appropriate. The search strategy is provided in Supplementary Table 1 and was validated using the PRESS guideline (30).

### Data extraction

Two reviewers (FL and PG) independently screened titles and abstracts of all studies that resulted from the search, reviewed full-text articles of all potentially relevant articles, and extracted data from eligible full-text articles. Discrepancies were resolved by discussion with a third reviewer (LS). Data collected included author, publication year, country, sex, age, CD diagnosis, PTSD/PTSS, index trauma, type of PTSD/PTSS measurement instruments, time of PTSD/PTSS measurement after CD diagnosis or initial treatment, sample size, and estimates. If results were based on the same study sample, the most comprehensive and recent publication was considered. For intervention studies with multiple measurement time points, baseline assessment data were included to evaluate cohorts prior to study interventions. To account for the sample dependence of multiple estimates within a study, the most recent eligible PTSD or PTSS assessment date for the prevalence or incidence estimate and the most commonly used measurement tool to collect PTSD or PTSS were extracted. If information or data were missing, the corresponding authors of the study were contacted and asked for further information.

### Assessment of study quality

Two researchers (FL and PG) independently assessed each included study for quality by using a risk of bias (RoB) appraisal instrument based on the Newcastle-Ottawa Scale (31) as well as the recommendations of the Cochrane Collaboration for the assessment of the risk of bias for non-comparative studies (32). This combined instrument addresses the quality of (1) sample representativeness, (2) prospective scheduling, (3) transparent, non-selected reporting of sample characteristics and outcomes, (4) sample size, (5) assessment, (6) data quality, and (7) comparability against a control group (full details regarding scoring provided in Supplementary Appendix 2). Studies were classified as having a high (1 to 3 points), moderate (4 to 6 points), or low (7 to 9 points) risk of bias. Discrepancies were resolved by discussion with a third reviewer (LS).

### Data synthesis and analysis

Prevalence and incidence of PTSD or PTSS in people with CD were calculated by pooling the study-specific estimates by using a generalized linear mixed model (GLMM). GLMM is an elaborate approach advocated especially for proportions (33). Since the effect sizes are based on continuous outcome data, the maximum-likelihood was used to estimate the GLMM (34). Prevalences and incidences are reported as percentages with corresponding 95% confidence interval (CI). Binary data were pooled as Odds Ratio (OR) by using the Bakbergenuly Sample Size Method (35) and the Paule-Mandel estimator was used to estimate the between-study variance (34). Bakbergenuly sample size method uses weights based on study-level sample size to estimate the overall effect, leading to a reduction in between-study variance bias than conventional methods (35). Estimates of PTSD or PTSS in people with CD versus without CD are reported as OR with corresponding 95% CI.

Forest plots were created to visually assess heterogeneity. Quantification of heterogeneity between studies was examined using the *I*^2^ statistic -as the ratio of variance between studies to total variance in the meta-analysis- and was tested for significance by using the *Q* statistics. A substantial level of heterogeneity was indicated by an *I*^2^ statistic value of 60% and greater (36). The larger *I*^2^, the greater the heterogeneity within the meta-analysis, because in it the scatter between the studies proportionally outweighs the random scatter within the studies (36). Records were defined as outliers if their CI did not match the CI of the pooled effect and differed significantly. This is because records with a high sampling error are likely to derivate significantly from the pooled effect (37). Potential moderators of prevalence and incidence estimates were calculated using random effects meta-regressions (36). Following the recommendation of Schwarzer et al. (38) and Borenstein et al. (39), meta-regression analysis was conducted in case of ≥10 studies per outcome (subgroup). From a number of ≥2 events per subcategory of the variable, the results of the meta-regressions are described descriptively (40, 41). The influence of each study on pooled estimate results was calculated using sensitivity analysis (leave one out analysis). Publication bias was investigated by funnel plot and Egger’s test (42).

All analyses were performed using the packages tidyverse, meta and metafor in the R software version 4.0.2 (43). Statistical tests were two-sided and used a significance threshold of *P* < 0.05. Changes to the *a priori* study registration were that in addition to narrative reviews, case reports and lack of full text availability, secondary literature such as meta-analyses, systematic reviews were excluded. However, the literature from the secondary literature was used for the additional manual search. In the case of substantial heterogeneity (>60%), data were pooled and presented with reports on heterogeneity and its interpretation. Possible reasons for the statistical heterogeneity were explored. In order to use a consistent method, meta-regressions were performed when there was substantial heterogeneity in the population (I^2^ > 60). Meta-regression analyses were performed in the case of ≥ 10 studies per outcome. The changes are shown in Supplementary Table 3.

## Results

### Study selection and characteristics

**FIGURE 1:**
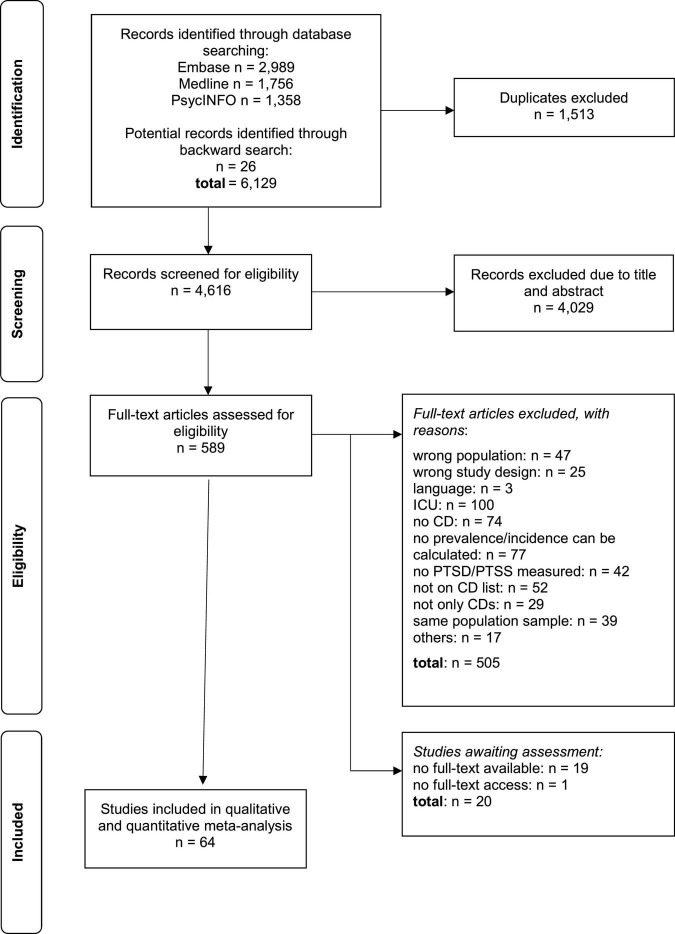
Selection process of primary studies.

A total of 6,103 references were identified through the electronic database searches and 26 additional studies through backward searches. After removal of duplicates, a total of 4,616 studies were reviewed; of these, 589 full-text articles were reviewed, and 64 were included in the systematic review. Reasons for exclusion of full texts were wrong population, wrong study design, language, intensive care unit, no CD or not on CD list, not only CD, no prevalence/incidence calculable, no PTSD/PTSS was measured, same population sample and no availability of full text and others (for PRISMA flow diagram see Figure 1).

Included studies were conducted in 17 different countries, predominantly in the USA (31 studies; 48%). 134 records from 60 references (*n* = 21,213) reported on the point prevalence of PTSD or PTSS; three records from three references (*n* = 913) reported on the 12-month prevalence of PTSD, and nine records of six references (*n* = 826) reported on the lifetime prevalence of PTSD. No references were found for the 12-month incidence of PTSD or PTSS in people with CD. The most commonly used questionnaires were Impact of Event Scale (Revised), PTSD Checklist and Post-traumatic Stress Diagnostic Scale. Structured Clinical Interview for DSM was the most frequently used structured interview. Nine different categorized CD were extracted (Tables 1, 2). Sample sizes of studies varied between 12 and 6,542 individuals. The mean age of individuals across studies was 41.14 years (SD = 8.72); 55.5% were female and 84.2% Caucasian. CD was diagnosed at a mean age of 21.52 years (SD = 15.64), from then on lasting a mean of 8.03 (SD = 4.58) years. With respect to study quality, four (6.3%) studies were at low, 53 (82.8%) at moderate, and seven (10.9%) at high risk of bias. A summary of selected study characteristics is presented in Supplementary Tables 4–6. See Supplementary Appendix 7 for references of included studies.

**TABLE 1:** Meta-regressions of point prevalence of PTSD in people with CD.

	Subgroup	*k*	Point-prevalence,%	95% CI	Estimate regression weight	95% CI	*I*^2^,%	*τ ^2^*	*QM*	*P*
PTSD	Age, mean	36			−0.01	−0.04 to 0.02	98.3	1.96	0.65	0.419
	Sex, female,%	40			0.00	−0.01 to 0.02	98.2	1.85	0.22	0.641
	Caucasian,%	22			−0.01	−0.04 to 0.03	98.2	1.59	0.19	0.660
	Risk of bias, sum score	41			−0.21	−0.60 to 0.17	97.8	1.81	1.17	0.280
	Publication year	41			−0.05	−0.14 to 0.04	98.0	1.72	1.13	0.287
	Timepoint of CD diagnosis	15			−0.02	−0.07 to 0.04	98.0	2.11	0.40	0.529
	Duration of CD	11			−0.02	−0.18 to 0.14	95.5	2.10	0.05	0.821
	Time of PTSD assessment, in years after CD diagnosis or initial CD treatment	29			−0.05	−0.11 to 0.02	97.5	1.83	2.16	0.142
	Type of PTSD instrument	41			0.87	−0.06 to 1.81	98.1	1.70	3.37	0.067
	Structured interview	12	7.0	2.5 to 18.0			93.5	3.32		
	Questionnaire	29	16.2	11.5 to 22.3			96.6	1.06		
	Type of CD	41			0.03	−0.08 to 0.14	98.1	1.84	0.29	0.593
	Cardiovascular diseases	5	6.6	1.9 to 20.9			90.7	1.87		
	Melignant neoplasm	17	12.8	6.6 to 23.3			95.6	2.19		
	Musculoskeletal disorders	5	8.5	1.8 to 32.1			97.4	3.35		
	Cerebrovascular disorders	4	23.6	16.8 to 31.9			66.5	0.11		
	Metabolic disorders	1	17.9				–	–		
	Nervous system diseases	7	15.9	7.6 to 30.4			91.7	1.14		
	Virus diseases	1	8.3				–	–		
	Lung diseases	1	22.5				–	–		
	Setting	40			−0.07	−0.26 to 0.12	98.1	1.85	0.52	0.469
	Inpatients	7	21.0	11.8 to 34.6			88.7	0.75		
	Outpatients	7	7.8	3.1 to 18.2			88.3	1.24		
	General population	17	13.1	6.7 to 23.9			96.2	2.29		
	Mixed sample	4	19.0	6.9 to 42.7			96.5	1.33		
	Other	5	8.7	2.2 to 28.5			93.4	2.47		
	Stage of CD	41			−0.22	−0.65 to 0.21	97.8	1.71	3.21	0.360
	Newly diagnosed	5	24.6	11.6 to 44.9				1.03	97.4	
	Progressive	2	20.6	14.6 to 28.2				0	0	
	Survivor	15	9.1	4.8 to 16.6				1.64	92.2	
	Mixed sample	19	12.8	6.8 to 22.9				2.24	94.9	

CD, chronic somatic disease; %, percentage; CI, confidence interval; estimate, model’s estimate of the regression weight; *I*^2^, residual heterogeneity; k, number of studies; *QM*, *Q*-test of moderators; *P*, *p*-value of the test of moderators; PTSD, post-traumatic stress disorder; *τ*^2^, estimate of the residual heterogeneity variance, the variance that is not explained by the predictor; *, statistically significant *p* < 0.05.

**TABLE 2:** Meta-regressions of point prevalence of PTSS in people with CD.

	Subgroup	*k*	Point-prevalence,%	95% CI	Estimate regression weight	95% CI	*I^2^,%*	*τ ^2^*	*QM*	*P*
PTSS	Age, mean	22			0.01	−0.02 to 0.04	97.6	1.4	0.42	0.516
	Sex, female,%	24			0.01	−0.01 to 0.03	97.5	1.2	0.89	0.345
	Caucasian,%	9			−0.02	−0.08 to 0.05	98.8	2.2	0.27	0.605
	Risk of bias, sum score	24			−0.53	−0.89 to −0.16	96.4	0.9	8.05	0.004*
	Publication year	24			0.00	−0.08 to 0.08	97.3	1.3	0.00	0.952
	Timepoint of CD diagnosis	11			0.00	−0.02 to 0.02	96.0	0.5	0.00	0.973
	Duration of CD	7			0.10	0.04 to 0.17	65.3	0.1	9.55	0.002*
	Time of PTSS assessment, in years after CD diagnosis or initial CD treatment	17			0.07	−0.03 to 0.16	97.0	1.6	1.89	0.161
	Type of PTSS instrument	24			2.56	0.79 to 4.32	98.9	0.9	8.06	0.005*
	Structured interview	2	2.7	1.0 to 6.9			0.0	0		
	Questionnaire	22	22.3	15.7 to 30.7			96.1	1.0		
	Type of CD		24		−0.02	−0.12 to 0.07	97.5	1.3	0.20	0.656
	Cardiovascular diseases	4	15.3	9.1 to 24.8			78.4	0.3		
	Melignant neoplasm	13	21.7	11.6 to 36.8			96.6	1.8		
	Musculoskeletal disorders	1	50.3				–	–		
	Metabolic disorders	3	13.1	2.4 to 48.2			88.7	2.3		
	Nervous system diseases	1	16.3				–	–		
	Infectious disease	1	9.4				–	–		
	Type 2 diabetes + cancer	1	30.4				–	–		
	Setting	21			−0.34	−0.82 to 0.14	97.3	1.2	1.90	0.167
	Inpatients	3	19.3	13.2 to 27.2			66.4	0.1		
	Outpatients	5	36.6	6.6 to 82.5			80.2	5.2		
	General population	10	19.3	13.2 to 27.4			97.3	0.5		
	Mixed sample	3	6.9	1.0 to 35.7			94.0	2.7		
	Stage of CD	24	19.3	0.25		−0.15 to 0.66	97.4	1.2	1.48	0.223
	Newly diagnosed	4	15.0	9.4 to 23.2			86.2	0.2		
	Progressive	4	8.7	2.5 to 26.1			84.8	1.5		
	Survivor	5	29.2	18.3 to 43.1			88.5	0.4		
	Mixed sample	11	23.3	11.5 to 41.6			97.1	2.0		

CD, chronic somatic disease; %, percentage; CI, confidence interval; estimate, model’s estimate of the regression weight; *I*^2^, residual heterogeneity; k, number of studies; *QM*, Q-test of moderators; *P*, *p*-value of the test of moderators; PTSS, post-traumatic stress symptoms; *τ*^2^, estimate of the residual heterogeneity variance, the variance that is not explained by the predictor; *, statistically significant *p* < 0.05.

### Point prevalence of PTSD or PTSS in people with CD

**FIGURE 2:**
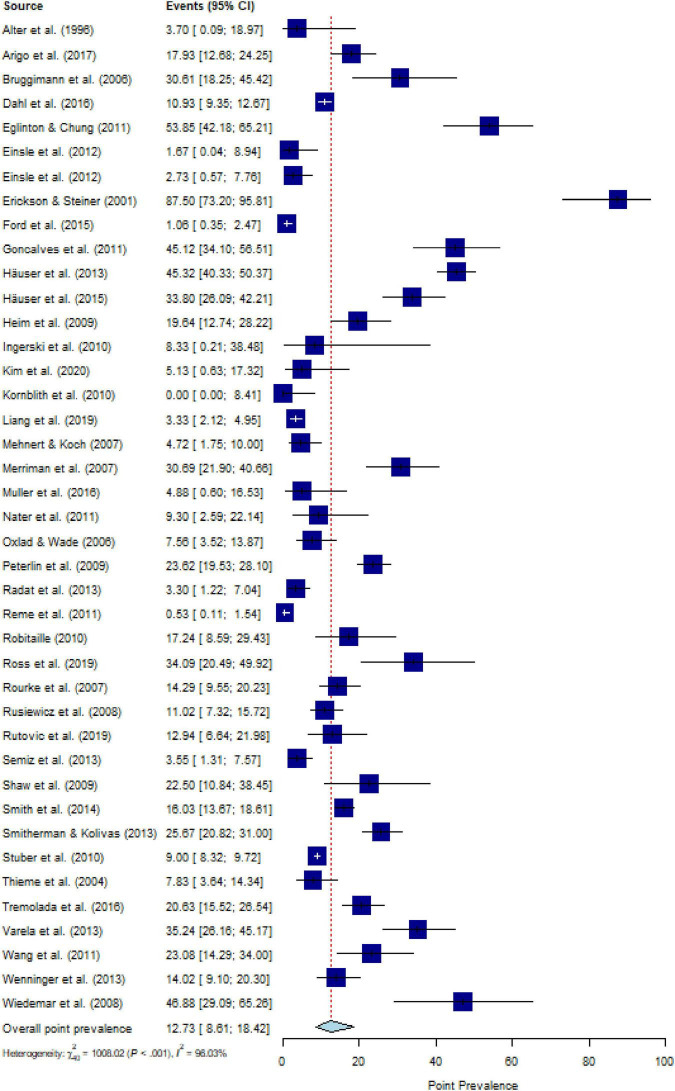
Forest plot of point prevalence of post-traumatic stress disorder (PTSD) in people with chronic somatic diseases (CD).

The most common measurement was self-report questionnaires (70.7% for PTSD, and 91.9% for PTSS); the remaining point prevalence estimates were collected by means of structured interviews. The duration of CD was 9.69 (SD = 6.08) years regarding PTSD and 5.36 (SD = 4.44) years regarding PTSS. The pooled estimate for the point prevalence of PTSD in people with CD [number of included studies (k) = 41] was 12.7% (95% CI, 8.6 to 18.4%), with substantial heterogeneity (*Q* = 1233.82, *P* < 0.001, *τ^2^* = 1.36, *I*^2^ = 96.0%) (Figure 2). After eliminating 18 outliers, heterogeneity was *I^2^* = 82.2% (*Q* = 130.57, *P* < 0.001, *τ^2^* = 0.25) and pooled estimate was 11.1% (95% CI, 8.8% to 14.0%). Sensitivity analysis (*k* = 41) demonstrated that no individual study affected the overall prevalence estimate of PTSD for more than 1% (Supplementary Figure 8).

**FIGURE 3:**
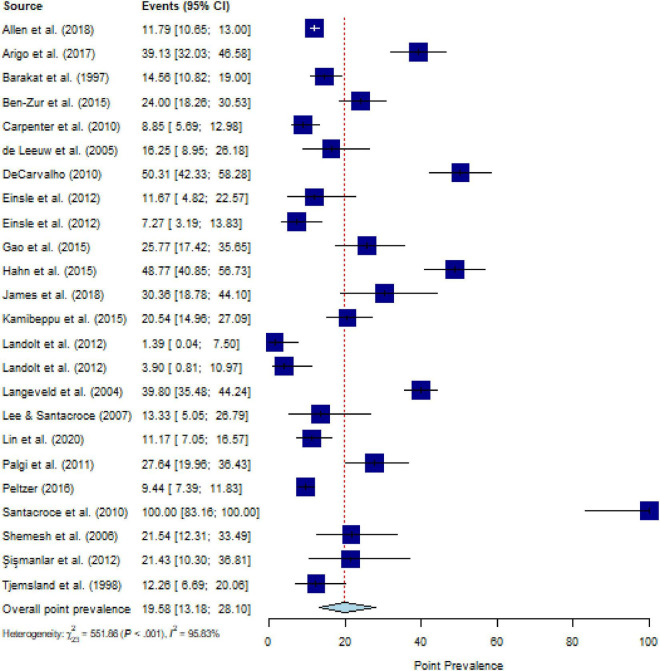
Forest plot of point prevalence of post-traumatic stress symptoms (PTSS) in people with chronic somatic diseases (CD).

The pooled estimate for the point prevalence of PTSS in people with CD (*k* = 24) was 19.6% (95% CI, 13.2 to 28.1%), with substantial heterogeneity (*Q* = 635.55, *P* < 0.001, *τ^2^* = 1.14, *I*^2^ = 95.8%) (Figure 3). After eliminating ten outliers, heterogeneity was *I^2^* = 70.2% (*Q* = 46.84, *P* < 0.001, *τ^2^* = 0.39) and pooled estimate was 17.6% (95% CI, 14.3% to 21.5%). Sensitivity analysis (*k* = 24) demonstrated that no individual study affected the overall prevalence estimate of PTSS for more than 2% (Supplementary Figure 9).

### Moderators of point prevalence of PTSD or PTSS in people with CD

Meta-regressions for point prevalence of categorized CDs comorbid to PTSD (*P* = 0.593) or PTSS (*P* = 0.656) did not yield significant results. The point prevalences for comorbid PTSD in CD subcategories range from 23.6% (95% CI, 16.8 to 31.9%) for cerebrovascular disorders (*k* = 4) to 6.6% (95% CI, 1.9 to 20.9%) for cardiovascular diseases (*k* = 5), with substantial heterogeneity (*Q* = 1112.62, *P* < 0.001, *τ^2^* = 1.83, *I*^2^ = 98.1%) (Table 1). The point prevalences for comorbid PTSS in CD subcategories range from 21.7% (95% CI, 11.6% to 36.8%) for malignant neoplasm (*k* = 13) to 13.1% (95% CI, 2.4% to 48.2%) for metabolic disorders (*k* = 3), with substantial heterogeneity (*Q* = 627.14, *P* < 0.001, *τ^2^* = 1.29, *I*^2^ = 97.5%). Descriptively, for comorbid PTSS, the highest prevalence of 50.3% was reported for musculosketal disorders (*k* = 1) and the lowest prevalence of 9.4% was reported for infectious disease (*k* = 1) (Table 2). Meta-regressions for point prevalence of PTSS in people with CD yielded a significant result for type of PTSS instrument [structured interview (*k* = 2) 2.7% (95% CI, 1.0 to 6.9%) vs. questionnaire (*k* = 22) 22.3% (95% CI, 15.7 to 30.7%; *P* = 0.005)], with substantial heterogeneity (*Q* = 601.85, *P* < 0.001, *τ^2^* = 0.93, *I*^2^ = 96.9%), for RoB sum score in PTSS studies [*k* = 24; *g* = −0.53 (95% CI, −0.89 to −0.16; *P* = 0.004)], with substantial heterogeneity (*QM* = 8.05, *τ^2^* = 0.96, *I*^2^ = 96.4%), and for duration of CD in PTSS studies [*k* = 7; *g* = 0.10 (95% CI, 0.04 to 0.17; *P* = 0.002)], with substantial heterogeneity (*QM* = 9.55, *τ^2^* = 0.09, *I*^2^ = 95.3%). These potential moderators were not a moderating factor for point prevalence in studies on PTSD, nor were the variables time of assessment of PTSD or PTSS in years after CD diagnosis or initial CD treatment, timepoint of CD diagnosis, stage of CD, age, setting, gender, ethnicity, and publication year, for both PTSD and PTSS (Table 1 for PTSD and Table 2 for PTSS).

### 12-month Prevalence of PTSD across CDs, and their moderators

**FIGURE 4:**
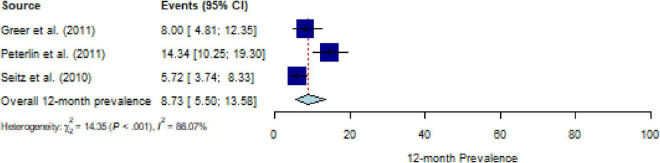
Forest plot of 12-month prevalence of post-traumatic stress disorder (PTSD) in people with chronic somatic diseases (CD).

All included studies assessed 12-month PTSD prevalence using a structured interview; studies assessing PTSS were not available. The pooled estimate for the 12-month prevalence in people with CD (*k* = 3) was 8.75% (95% CI, 5.5% to 13.5%), with substantial heterogeneity (*Q* = 14.20, *P* < 0.001, *τ^2^* = 0.38, *I*^2^ = 86.1%) (Figure 4). No outliers were detected. The number of studies was too small (≤ 10 studies) to calculate meta-regressions. Descriptively, an estimate for specific CDs of 14.3% for nervous system diseases (*k* = 1) and a weighted mean of 6.5% for malignant neoplasms (*k* = 2) can be reported.

### Lifetime prevalence of PTSD across CDs, and their moderators

**FIGURE 5:**
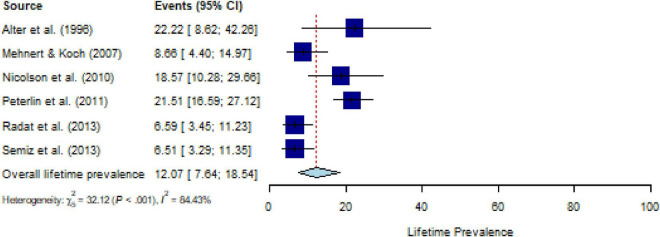
Forest plot of lifetime prevalence of post-traumatic stress disorder (PTSD) in people with chronic somatic diseases (CD).

All studies assessed lifetime PTSD prevalence using a structured interview; studies assessing PTSS were not available. The pooled estimate for lifetime prevalence of PTSD in people with CD (*k* = 6) was 12.1% (95% CI, 7.6 to 18.5%), with substantial heterogeneity (*Q* = 35.02, *P* < 0.001, *τ^2^* = 0.30, *I*^2^ = 84.4%) (Figure 5). After eliminating one outlier, the heterogeneity was *I^2^* = 72.3% (*Q* = 13.21, *P* < 0.01, *τ^2^* = 0.45), and the pooled estimate was 10.2% (95% CI, 6.4 to 15.7%). Sensitivity analysis (*k* = 6) demonstrated that no individual study affected the prevalence estimate of PTSD for more than 2% (Supplementary Figure 10). The number of studies was too small (≤ 10 studies) to calculate meta-regressions. Descriptively, weighted means for comorbid PTSD and specific CDs showed a lifetime prevalence of 11.0% for malignant neoplasms (*k* = 2), 9.9% for musculoskeletal disorders (*k* = 2), and 15.5% for nervous system diseases (*k* = 2).

### PTSD in people with CD compared to controls without CD

**FIGURE 6:**
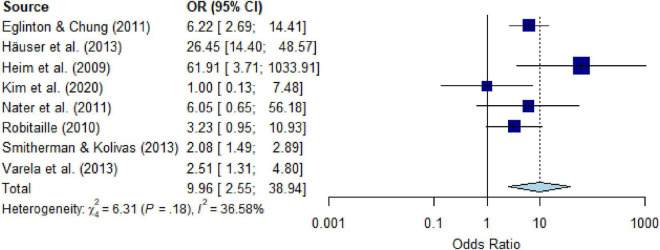
Forest plot of point prevalence of post-traumatic stress disorder (PTSD) in people with chronic somatic diseases (CD) compared with controls without chronic somatic diseases (CD), after eliminating outliers.

All studies on the comparison between PTSD or PTSS in people with versus those without CD assessed PTSD, with 87.5% using self-report questionnaires; studies assessing PTSS were not available. Meta-analytic pooling (*k* = 8) yielded an OR of 7.09 (95% CI, 2.49 to 20.17) (Supplementary Figure 11), with substantial heterogeneity *I^2^* = 88.4% (*Q* = 60.51, *P* < 0.001, *τ^2^* = 10.05). After eliminating five outliers (*k* = 3) the heterogeneity decreased to a moderate *I^2^* = 36.6% (*Q* = 6.31, *P* = 0.178, *τ^2^* = 0.71) and the estimate yielded an OR of 9.96 (95% CI, 2.55 to 38.94) (Figure 6). The number of studies was too small (≥ 10 studies) to calculate meta-regressions. Descriptively, people with versus those without specific CD showed increased risk for PTSD of OR = 2.51 and OR = 3.23 for malignant neoplasm (*k* = 2), OR = 26.45 for musculoskeletal disorders (*k* = 1), and OR = 2.08 to 61.91 for nervous system diseases (*k* = 5).

### Publication bias

The Egger’s test for point prevalence estimates of PTSD (*Intercept* = 0.91 (95% CI, −1.4% to −3.2%, *P* = 0.45) (Supplementary Figure 12) and PTSS (*Intercept* = 0.78 (95% CI, −2.7% to −4.3%, *P* = 0.67) (Supplementary Figure 13) in people with CD does not indicate the presence of funnel plot asymmetry and thus publication bias. The number of studies of 12-month and lifetime prevalence of PTSD in people with CD were too small to test for small-study effects with Egger’s test assessing publication bias.

## Discussion

People with CD show seven to ten times increased odds for PTSD compared to people without CD. This impressive number is matched by a pooled point prevalence of 12.7% for PTSD and 19.6% for PTSS in people with CD. Moreover, PTSS and PTSD seem to represent a particularly increased comorbidity risk factor for people with CD, given substantially lower OR reported for other mental disorders such as depression (OR = 1.3), anxiety (OR = 1.6), and somatoform disorders (OR = 0.9) in people with versus without CD (44). The results of the present systematic review are supported by estimates of the World Mental Health Surveys (45), indicating a point prevalence of PTSD in the general population of about one in seventy-one persons (1.4%) compared to about one in five (12.7%), as shown here.

The apparent finding that the point prevalence of PTSS in people with CD was higher compared to PTSD is an inherent part of the construct definitions. For PTSD, more clinical symptoms must be present to fulfill the criteria of DSM or ICD classification systems than for elevated PTSS, even though both manifestations lead to symptom worsening of the CD, reduced treatment adherence, psychological distress, functional impairment (14–18), poor quality of life (46), and long-term health decline (47). Furthermore, PTSD was predominantly assessed by structured interviews, while PTSS was mainly assessed by means of questionnaires, with the latter being associated with higher prevalence rates than the former (23). These methodological factors need to be considered when interpreting the prevalence rates of PTSS and PTSD.

Interestingly, prevalence rates for PTSD (6.6 to 23.6%) and PTSS (9.4 to 50.3%) varied widely across CD, while however, non-significantly. Due to known differences in disease severity, different treatment methods (25), remission or improvement prospects, disease impairments, the mental adjustment to CD, and rehabilitation (48), these non-significant results may rather be interpreted methodologically, given the small number of studies per subcategory, the lack of studies for certain CDs such as gastrointestinal, skin, or kidney diseases, and varying methods of assessing PTSD and PTSS. Prevalences of specific CD categories and their moderators could provide additional information about specific risk groups. For example, there was a significant increase in the point-prevalence of PTSS over the duration of CD. Additionally, people with CD and comorbid PTSD had a longer average duration of CD than those with CD and comorbid PTSS. Assuming that PTSS could lead to PTSD if it worsens, and that PTSS can worsen with the duration of CD, the longer average duration of CD in PTSD compared to PTSS could be an indication of a temporal association between symptom worsening with longer duration of CD. Swartzman et al. (25) found opposite results for the duration of cancer in the sense of a symptom reduction. Nevertheless, overall the data suggest that duration of CD is associated with symptom severity of PTSS and PTSD. For clinical practice, this would mean that mental health screening is important at both initial diagnosis and during the course of CD. Hence, there remains a long way of scientific effort for unraveling the prevalence rates and the differential impact of different CD on PTSD and PTSS, with consideration of symptom severity longitudinally.

Further moderator analyses, exclusion of outliers, and sensitivity analyses could not resolve the substantial heterogeneity of studies addressing the prevalence of PTSD or PTSS in CD; therefore, prevalence estimates should be considered with caution. The differences in prevalence could have resulted from different study designs with different levels of methodological quality, study populations, sample sizes, sampling methods, data collection, and collaboration of study participants. Furthermore, demographic protective factors, such as high level of education, paired relationship or being married, being employed, higher economic status, and social support seem to play a role in the occurrence of PTSD or PTSS in CD (49, 50), and could explain heterogeneity. Also, the dose-response effect of traumatic stress could have an impact on heterogeneity, as a higher number of different lifetime traumatic event types (i.e., more intense traumatization) were associated with a higher probability of point and lifetime PTSD and with a reduced probability of long-term spontaneous remission from PTSD (51). Moreover, the assessment approach, in which only CD as index trauma is examined compared to any index trauma, could have a substantial impact on PTSD and PTSS prevalence (52). By including the studies with different methodological approaches, a limitation of missing data can be reduced. Qualitative statements on the relevance of the topic can only be made on the basis of data pooling. Purely descriptive presentations of results could mean that many new results are not recognized. In order to be able to meta-analyze the heterogeneity with the current data situation, the variables mentioned would have to have been collected in the primary studies. These factors explaining the heterogeneity are of interest for future research.

The distinctive feature of PTSD compared to other mental diseases is its explainable etiology (4, 53). A CD diagnosis with increased mortality, its possible worsening, treatment, and challenging behavioral and cognitive-emotional responsibilities (e.g., coping with a diagnosis or adhering to complex treatment schedules) are disease-related distressors (48) that may traumatize (3, 24, 54, 55). PTSD has a negative impact on CD medication and treatment adherence, especially if the PTSD was induced by the CD with increased mortality or a related medical event. CD treatment can serve as an aversive reminder and reinforce the avoidance behavior characteristic of PTSD (24, 56, 57). In addition to the etiological association, PTSD is associated with poorer health behavior, which may be risk factors for developing CD and may negatively affect CD outcome (58–60). These bidirectional associations and the increased risk of comorbidity for PTSD in people with CD could be given more attention in clinical practice to support the mental and physical health of people with CD.

Incidences of PTSD in people with CD might point to the etiological association. However, analysis of 12-month incidences was not possible given the lack of available primary studies. Prospective studies of comorbid CD and PTSD often address the incidence of CD in people with PTSD (58). Hereby, PTSD was associated with the onset of (self-reported) CD in a dose-response ratio. Women with the highest number of PTSD symptoms had a nearly two-fold increased risk of type 2 diabetes mellitus compared with women without PTSD or PTSS (58). Twice the incidence of PTSD compared to twins without PTSD was also found for other CD, such as coronary heart disease (59). These findings indicate a bidirectional relationship between PTSD and CD, suggesting a comprehensive medical history, including a psychosocial one, in the treatment of CD. Especially since the early diagnostics and treatment of PTSD in CD, in addition to reducing PTSD (61, 62), leads to a decrease of depressive symptoms (63), sleep problems, various chronic somatic health complaints such as back pain, cough (64), and a reduction of hypertension (65) and cardiovascular risk (66).

### Limitations

When interpreting the results some limitation of our systematic review and meta-analysis should be considered. Random error is a general problem despite the total number of 22,952 subjects on which the analysis was based. Access to the original study data and thus to epidemiologic data was not available. Study populations were found mainly from industrialized nations and only German or English language studies were included, which limits the world-wide generalizability. Although this meta-analysis included different categories of CD, most studies included people with malignant neoplasms, cardiovascular diseases, musculoskeletal disorders or nervous system diseases, so the results are dominated by these CD categories. Causal attributions between CD and PTSD or PTSS cannot be drawn based on the data.

## Conclusion

The results suggest that PTSD is a common mental health comorbidity in people with CD. The very high OR for PTSD in people with CD compared to people without CD suggest a specific link between PTSD and CD beyond the generic fact of increased mental burden in people with CD. Systematic early PTSD screening in people with CD may facilitate identification of individuals in need of support. Earlier detection and treatment of comorbid PTSD or PTSS in people with CD might provide a means to improve health outcomes, treatment adherence, and quality of life (62, 67). Moreover, we should increase awareness for this specific comorbidity in somatic health care, as clinical practice and treatment guidelines today are mainly focused on depression as comorbidity (68, 69).

## Data availability statement

The original contributions presented in this study are included in this article/Supplementary material, further inquiries can be directed to the corresponding author.

## Author contributions

FL and PG conducted the systematic literature searches, extracted the data, rated the data and the risk of bias of included studies, had full access of the data in the study, and take responsibility for the integrity of the data. LS resolved discrepancies. FL analyzed the data and wrote the first draft of the manuscript. All authors have contributed to the design of the study, writing, and approved the final manuscript.
