# Supplementary material for: Prevalence and incidence of post-traumatic stress disorder and symptoms in people with chronic somatic diseases: A systematic review and meta-analysis
Source: Front Psychiatry. 2023 Jan 18;14:1107144. doi: 10.3389/fpsyt.2023.1107144 (PMC9889922; doi:10.3389/fpsyt.2023.1107144)
Supplement: Supplementary file 1 [file Data_Sheet_1.ZIP › S1. Search String.docx]

Supplementary table S1

Search strings for MEDLINE, Embase, and PsycINFO via Ovid MEDLINE Embase PsycINFO

|  | [chronic somatic disease] | | |
| --- | --- | --- | --- |
| **1** | exp Chronic Disease/ | exp chronic disease/ | exp Chronic Illness/ |
| **2** | Critical Illness/ | critical illness/ | critical illness.mp. |
| **3** | ((chronic* OR persistent OR incurable OR lifelong) ADJ2 (disease* OR condition* OR disorder* OR ill* OR problem* OR health)).ti,ab. | ((chronic* OR persistent OR incurable OR lifelong) ADJ2 (disease* OR condition* OR disorder* OR ill* OR problem* OR health)).ti,ab. | ((chronic* OR persistent OR incurable OR lifelong) ADJ2 (disease* OR condition* OR disorder* OR ill* OR problem* OR health)).ti,ab. |
| **4** | ((chronic* OR persistent OR incurable OR lifelong) ADJ2 (somatic* OR physic* OR medical*)).ti,ab. | ((chronic* OR persistent OR incurable OR lifelong) ADJ2 (somatic* OR physic* OR medical*)).ti,ab. | ((chronic* OR persistent OR incurable OR lifelong) ADJ2 (somatic* OR physic* OR medical*)).ti,ab. |
| **5** | (long term ADJ2 (condition* OR ill* OR disease* OR problem* OR disorder* OR health)).ti,ab. | (long term ADJ2 (condition* OR ill* OR disease* OR problem* OR disorder* OR health)).ti,ab. | (long term ADJ2 (condition* OR ill* OR disease* OR problem* OR disorder* OR health)).ti,ab. |
| **6** | (long term ADJ2 (somatic* OR physic* OR medical*)).ti,ab. | (long term ADJ2 (somatic* OR physic* OR medical*)).ti,ab. | (long term ADJ2 (somatic* OR physic* OR medical*)).ti,ab. |
| **7** | 3 AND 4 | 3 AND 4 | 3 AND 4 |
| **8** | 5 AND 6 | 5 AND 6 | 5 AND 6 |
| **9** | 7 OR 8 | 7 OR 8 | 7 OR 8 |

|  | **MEDLINE** | **Embase** | **PsycINFO** |
| --- | --- | --- | --- |
|  | [CD event] | | |
| **10** | medical events.mp. | medical events.mp. | medical event*.mp. |
| **11** | ((medical OR critical) ADJ2 (event* OR occasion OR occurrence OR incident)).ti,ab. | ((medical OR critical) ADJ2 (event* OR occasion OR occurrence OR incident)).ti,ab. | ((medical OR critical) ADJ2 (event* OR occasion OR occurrence OR incident)).ti,ab. |
| **12** | 1 OR 2 OR 9 OR 10 OR  11 | 1 OR 2 OR 9 OR 10 OR  11 | 1 OR 2 OR 9 OR 10 OR  11 |
|  | [Broader terms of specific chronic somatic diseases] | | |
| **13** | Cardiovascular Diseases/ | cardiovascular disease/ | Cardiovascular Disorders/ |
| **14** | Cerebrovascular Disorders/ | cerebrovascular disease/ | Cerebrovascular Disorders/ |
| **15** | Respiration Disorders/ | breathing disorder/ | Respiratory Tract Disorders/ |
| **16** | Lung Diseases/ | lung disease/ | Lung Disorders/ |
| **17** | Liver Diseases/ | liver disease/ | Liver Disorders/ |
| **18** | Musculoskeletal Diseases/ | musculoskeletal disease/ | Musculoskeletal Disorders/ |
| **19** | Joint Diseases/ | arthropathy/ | Joint Disorders/ |
| **20** | Connective Tissue Diseases/ | connective tissue disease/ | connective tissue disorders.mp. |
| **21** | Spinal Diseases/ | spine disease/ | spinal disorders.mp. |
| **22** | Bone Diseases/ | bone disease/ | Bone Disorders/ |
| **23** | Kidney Diseases/ | kidney disease/ | Kidney Diseases/ |
| **24** | Autoimmune Diseases/ | autoimmune disease/ | Immunologic Disorders/ |
| **25** | Skin Diseases/ | skin disease/ | Skin Disorders/ |
| **26** | Metabolic Diseases/ | metabolic disorder/ | Metabolism Disorders/ |

|  | **MEDLINE** | **Embase** | **PsycINFO** |
| --- | --- | --- | --- |
| **27** | Endocrine System Diseases/ | endocrine disease/ | - |
| **28** | Brain Diseases/ | brain disease/ | Brain Disorders/ |
| **29** | Nervous System Diseases/ | neurologic disease/ | Nervous System Disorders/ |
| **30** | Headache Disorders/ | “headache and facial pain”/ | Migraine Headache/ |
| **31** | Neuromuscular Diseases/ | neuromuscular disease/ | Neuromuscular Disorders/ |
| **32** | Neurodegenerative Diseases/ | degenerative disease/ | Neurodegenerative Diseases/ |
| **33** | Virus Diseases/ | virus infection/ | Viral Disorders/ |
| **34** | Ear Diseases/ | ear disease/ | Ear Disorders/ |
| **35** | Vascular Diseases/ | vascular disease/ | - |
| **36** | Heart Diseases/ | heart disease/ | Heart Disorders/ |
| **37** | Gastrointestinal Diseases/ | gastrointestinal disease/ | Gastrointestinal Disorders/ |
| **38** | Muscular Diseases/ | muscle disease/ | Muscular Disorders/ |
| **39** | Urologic Diseases/ | urinary tract disease/ | Urogenital Disorders/ |
| **40** | Basal Ganglia Diseases/ | extrapyramidal syndrome/ | - |
| **41** | Migraine Disorders/ | migraine/ | - |
| **42** | Sleep Wake Disorders/ | sleep disorder/ | Sleep Wake Disorders/ |
| **43** | Vestibular Diseases/ | vestibular disorder/ | Labyrinth Disorders/ |
| **44** | Neoplasms/ | neoplasm/ | Neoplasms/ |

|  | **MEDLINE** | **Embase** | **PsycINFO** |
| --- | --- | --- | --- |
| **45** | 13 OR 14 OR 15 OR 16  OR 17 OR 18 OR 19 OR  20 OR 21 OR 22 OR 23  OR 24 OR 25 OR 26 OR  27 OR 28 OR 29 OR 30  OR 31 OR 32 OR 33 OR  34 OR 35 OR 36 OR 37  OR 38 OR 39 OR 40 OR  41 OR 42 OR 43 OR 44 | 13 OR 14 OR 15 OR 16  OR 17 OR 18 OR 19 OR  20 OR 21 OR 22 OR 23  OR 24 OR 25 OR 26 OR  27 OR 28 OR 29 OR 30  OR 31 OR 32 OR 33 OR  34 OR 35 OR 36 OR 37  OR 38 OR 39 OR 40 OR  41 OR 42 OR 43 OR 44 | 13 OR 14 OR 15 OR 16  OR 17 OR 18 OR 19 OR  20 OR 21 OR 22 OR 23  OR 24 OR 25 OR 26 OR  28 OR 29 OR 30 OR 31  OR 32 OR 33 OR 34 OR  36 OR 37 OR 38 OR 39  OR 42 OR 43 OR 44 |
| **46** | (chronic* OR persistent OR incurable OR lifelong OR long-term).ti,ab. | (chronic* OR persistent OR incurable OR lifelong OR long-term).ti,ab. | (chronic* OR persistent OR incurable OR lifelong OR long-term).ti,ab. |
| **47** | 45 AND 46 | 45 AND 46 | 45 AND 46 |
| **48** | 12 OR 47 | 12 OR 47 | 12 OR 47 |
|  | [PTSD/PTSS] | | |
| **49** | exp Stress Disorders, Post- Traumatic/ | exp posttraumatic stress disorder/ | exp Posttraumatic Stress Disorder/ |
| **50** | Psychological Trauma/ | exp psychotrauma/ | exp Emotional Trauma/ |
| **51** | - | - | exp Post-Traumatic Stress/ |
| **52** | (post-trauma* ADJ2 stress).ti,ab. | (post-trauma* ADJ2 stress).ti,ab. | (post-trauma* ADJ2 stress).ti,ab. |
| **53** | (emotional ADJ2 trauma).ti,ab. | (emotional ADJ2 trauma).ti,ab. | (emotional ADJ2 trauma).ti,ab. |
| **54** | (post-trauma* ADJ2 symptom*).ti,ab. | (post-trauma* ADJ2 symptom*).ti,ab. | (post-trauma* ADJ2 symptom*).ti,ab. |
| **55** | (posttrauma* ADJ2 symptom*).ti,ab. | (posttrauma* ADJ2 symptom*).ti,ab. | (posttrauma* ADJ2 symptom*).ti,ab. |
| **56** | (posttrauma* ADJ2 stress).ti,ab. | (posttrauma* ADJ2 stress).ti,ab. | (posttrauma* ADJ2 stress).ti,ab. |

|  | **MEDLINE** | **Embase** | **PsycINFO** |  |
| --- | --- | --- | --- | --- |
| **57** | (psych* ADJ2 trauma).ti,ab. | (psych* ADJ2 trauma).ti,ab. | (psych* ADJ2 trauma).ti,ab. |  |
| **58** | (post-emotional ADJ2 stress).ti,ab. | (post-emotional ADJ2 stress).ti,ab. | (post-emotional ADJ2 stress).ti,ab. |  |
| **59** | ptsd.ti,ab. | ptsd.ti,ab. | ptsd.ti,ab. |  |
| **60** | | 49 OR 50 OR 52 OR 53  OR 54 OR 55 OR 56 OR  57 OR 58 OR 59 | 49 OR 50 OR 52 OR 53  OR 54 OR 55 OR 56 OR  57 OR 58 OR 59 | 49 OR 50 OR 51 OR 52  OR 53 OR 54 OR 55 OR  56 OR 57 OR 58 OR 59 |
| **61** | | 48 AND 60 | 48 AND 60 | 48 AND 60 |
