# Supplementary material for: Prevalence and incidence of post-traumatic stress disorder and symptoms in people with chronic somatic diseases: A systematic review and meta-analysis
Source: Front Psychiatry. 2023 Jan 18;14:1107144. doi: 10.3389/fpsyt.2023.1107144 (PMC9889922; doi:10.3389/fpsyt.2023.1107144)

**Supplementary figure S11. Forest plot of point prevalence of PTSD in people with CD compared with controls without CD.**


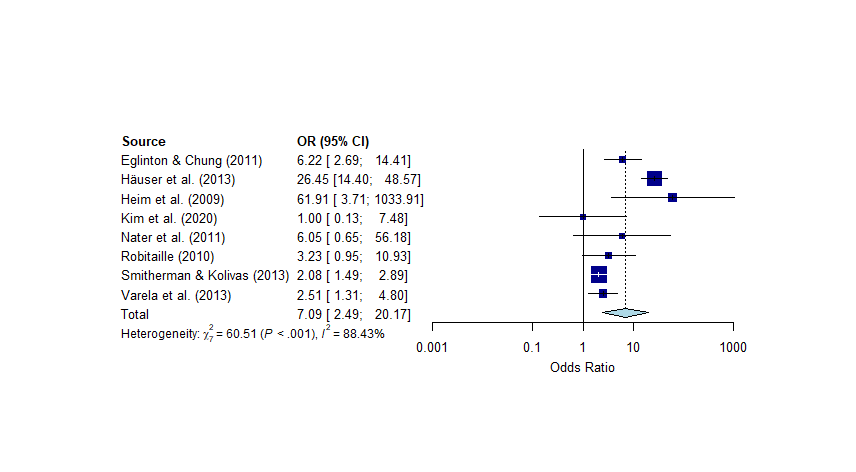

Supplement: Supplementary file 1 [file Data_Sheet_1.ZIP › S11. Forest Plot of Point Prevalence of PTSD compared with Controls.docx]
