# Supplementary material for: Prevalence and incidence of post-traumatic stress disorder and symptoms in people with chronic somatic diseases: A systematic review and meta-analysis
Source: Front Psychiatry. 2023 Jan 18;14:1107144. doi: 10.3389/fpsyt.2023.1107144 (PMC9889922; doi:10.3389/fpsyt.2023.1107144)
Supplement: Supplementary file 1 [file Data_Sheet_1.ZIP › S13. Publication Bias of the Point Prevalence of PTSS.docx]

**Supplementary figure S13. Publication Bias of the Point Prevalence of PTSS**

**Assessment of small-study effects of point prevalence estimates of PTSS by funnel plot and Egger´s test.**


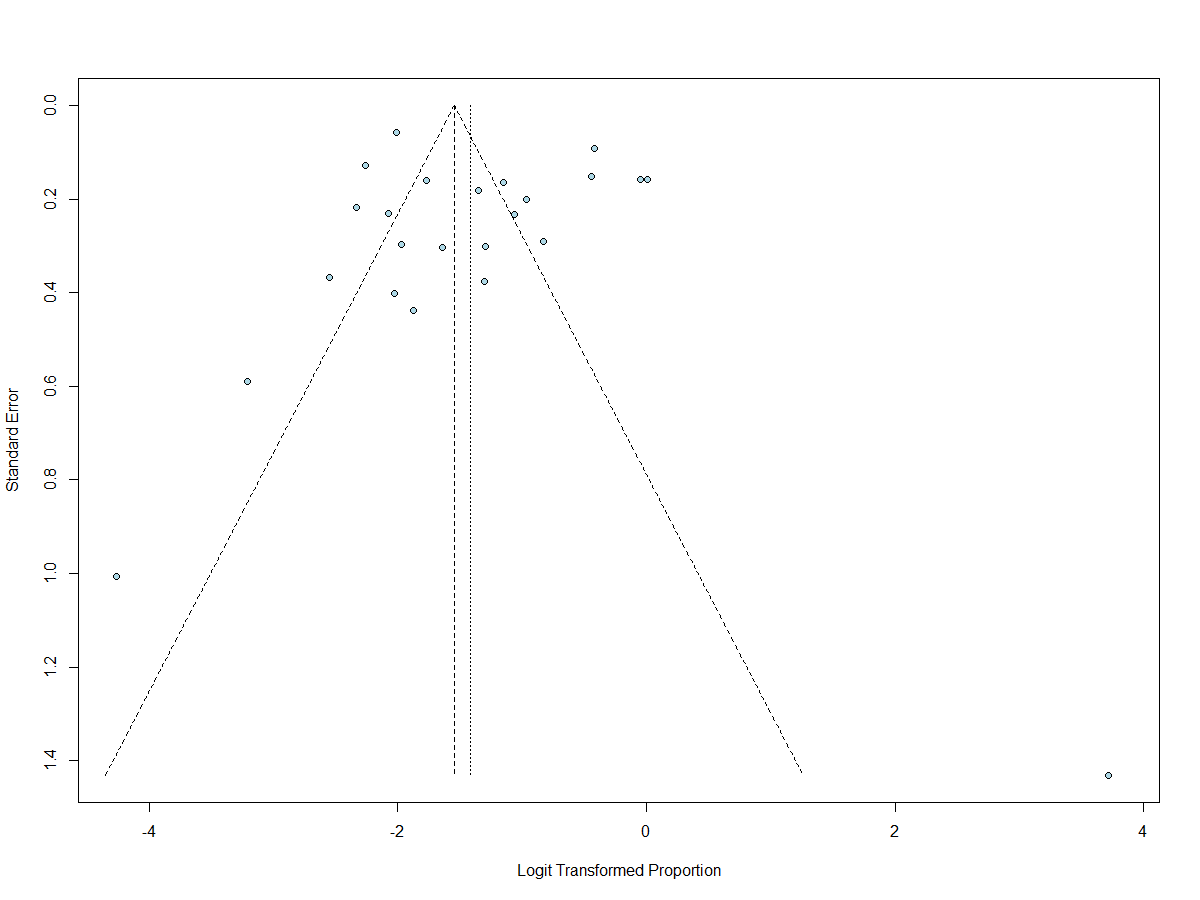


**Legend:** The linear regression test of funnel plot asymmetry does not indicate the presence of publication bias (*P* = .67).
