# Supplementary material for: Prevalence and incidence of post-traumatic stress disorder and symptoms in people with chronic somatic diseases: A systematic review and meta-analysis
Source: Front Psychiatry. 2023 Jan 18;14:1107144. doi: 10.3389/fpsyt.2023.1107144 (PMC9889922; doi:10.3389/fpsyt.2023.1107144)
Supplement: Supplementary file 1 [file Data_Sheet_1.ZIP › S2. Risk of Bias Assessment Tool.docx]

**Supplementary Appendix S2**

**Risk of bias assessment tool**

(1) Sample representativeness

*1 point:* Yes or likely yes (e.g. consecutive; all cases in a certain area or certain time period).

*0 points:* No (selection bias is likely or no (sufficient) information is given).

(2) Prospective scheduling

*1 point:* A study protocol is available with predefined inclusion criteria and outcomes.

*0 points:* No study protocol or no (sufficient) information is given.

(3) Transparent, non-selected reporting of sample characteristics and outcomes

*1 point:* The study reports descriptive statistics to describe the population and reports all outcomes.

*0 points:* The study does not report or reports incomplete or selective sample characteristics and outcomes.

(4) Sample size

*1 point:* Sample size is greater than or equal to 200 participants.

*0 points:* Sample size is less than 200 participants.

(5) Assessment of PTSD or PTSS and chronic somatic disease

*2 points:* The study uses a standardised measurement tool (validated interview, questionnaire, or screening; or a standardised international classification system) for assessing PTSD or PTSS and chronic somatic disease.

*1 point:* Assessing either PTSD/PTSS or chronic somatic disease with a standardised measurement tool. The other one was assessed with a non-standardised measurement tool.

*0 points:* The study used non-standardised measurement tools for both PTSD/ PTSS and chronic somatic disease or no (sufficient) information is given.

(6) Data quality (with regard to treating missing values)

*2 points:* Missing data is adequately taken into account in the statistical analyses (e.g. full information maximum likelihood, multiple imputation).

*1 point:* Missing data is taken into account but a weak missing data approach is used (e.g. listwise deletion, pairwise deletion, single imputation).

*0 points:* Missing data is not taken into account or no (sufficient) information is given.

(7) Comparability against a control group

*1 point:* Presence of a control group for comparing epidemiologic estimates.

*0 points:* No control group.

*Legend*. The components listed above generate a total sum score for assessing risk of bias for each study. Total sum scores range from 0 to 9 points. Studies were grouped into ‘low risk of bias’ (7-9 points), ‘moderate risk of bias’ (4-6 points) or ‘high risk of bias’ (1-3 points).
