# Supplementary material for: Prevalence and incidence of post-traumatic stress disorder and symptoms in people with chronic somatic diseases: A systematic review and meta-analysis
Source: Front Psychiatry. 2023 Jan 18;14:1107144. doi: 10.3389/fpsyt.2023.1107144 (PMC9889922; doi:10.3389/fpsyt.2023.1107144)
Supplement: Supplementary file 1 [file Data_Sheet_1.ZIP › S3. Changes to the a-priori study registration.docx]

**Supplementary table S3. Changes to the a-priori study registration**

| **a-priori study registration** | **finale methodical realization** |
| --- | --- |
| *Exclusion:* narrative reviews, case reports, no full text available | *Exclusion:* narrative reviews, case reports, no full text available and secondary literature: meta-analysis, systematic reviews. From reviews and meta-analyses, the included literature was used in the additional manual search. |
| Data analyses will be performed using Review Manager 5.3. | All analyses were performed using the software R version 4.0.2. |
| Only studies showing less than substantial statistical heterogeneity (0-60%) will be pooled. In case of substantial statistical heterogeneity, we will abstain from meta-analytical pooling of outcomes across studies and will only report the range of prevalence and incidence rates, descriptively summarize the evidence and explore potential reasons for statistical heterogeneity. | In cases of substantial heterogeneity (>60%), data were pooled and presented with the reports of heterogeneity and their interpretation. Potential reasons for statistical heterogeneity were explored. |
| If possible, meta-regressions will be calculated for continuous predictors in a random effects model. If there is a substantial heterogeneity in the population sample (I² > 60) and categorial predictors, we will perform subgroup analyses, in order to identify potential sources that can explain the substantial heterogeneity.  Subgroup analyses will be conducted if at least three studies deal with the respective categories. | To use a consistent method, substantial heterogeneity in the population sample ((I² > 60) were performed by meta-regressions. Meta-regression analysis were conducted in case of >= 10 studies per outcome. |
