# Supplementary material for: Prevalence and incidence of post-traumatic stress disorder and symptoms in people with chronic somatic diseases: A systematic review and meta-analysis
Source: Front Psychiatry. 2023 Jan 18;14:1107144. doi: 10.3389/fpsyt.2023.1107144 (PMC9889922; doi:10.3389/fpsyt.2023.1107144)
Supplement: Supplementary file 1 [file Data_Sheet_1.ZIP › S4. Study characteristics point prevalence.docx]

| **Article label** | **Country** | **Age, mean (SD)** | | **Female, %** | | **Caucasian, %** | **Name of CD** | **Stage of CD** | **Duration of CD, years** | **Timepoint diagnosis CD, years** | **Time of PTSS/ PTSD assesment, years after diagnosis** | **PTSD instrument** | **Cut-off Score** | **PTSS/ PTSD cases** | **Total** | **RoB ranking** |
| --- | --- | --- | --- | --- | --- | --- | --- | --- | --- | --- | --- | --- | --- | --- | --- | --- |
| Allen et al. 2018 | USA | 32.5 (8.5) | 49.1 | | 85.1 | | cancer | survivor | NA | 8.4 | 24.1 | PCL-C | 44 | 350 | 2969 | 2 |
| Alter et al. 1996 | USA | 54.0 (11.0) | 100 | | 100 | | cancer | survivor | 5.4 | NA | 5.4 | SCID | NA | 1 | 27 | 2 |
| Arigo et al. 2017 | USA | 55.4 (10.17) | 81 | | 81 | | diabetes mellitus type 2 | progressive | 11.99 | 42.7 | 12 | IES-R | 19 | 72 | 184 | 2 |
| Barakat et al. 1997 | USA | 13.5 (3.4) | 50 | | 87 | | cancer | survivor | NA | 5.83 | NA | UCLA PTSD RI | NA | 45 | 309 | 2 |
| Ben-Zur et al. 2015 | Israel | 62.5 (6.2) | 42.5 | | NA | | cancer | survivor | 2.07 | 60.45 | NA | PSS | NA | 48 | 200 | 2 |
| Bruggiman et al. 2006 | Switzerland | 51.4 (16.2) | 32.65 | | NA | | ischemic stroke, hemorrhagic stroke | survivor | NA | NA | 1 | IES | 30 | 15 | 49 | 2 |
| Carpenter et al. 2010 | USA | 56.4 (12.3) | 100 | | 95 | | cancer | survivor | NA | 52.13 | 4.3 | IES-R | 33 | 23 | 260 | 2 |
| Dahl et al. 2016 | Norway | 46.5 (11.6) | 0 | | NA | | cancer | survivor | NA | 35.0 | 11 | IES | 35 | 155 | 1418 | 2 |
| de Leeuw et al. 2005 | USA | 37.1 (11.7) | 78.75 | | NA | | migraine, tension-type headache | newly diagnosed, progressive | 5.57 | NA | NA | PCL-C | 41 | 13 | 80 | 2 |
| DeCarvalho 2010 | USA | 45.3 (15.1) | 63.35 | | 74.5 | | CLBP | progressive | 11 | NA | NA | PDS | NA | 81 | 161 | 2 |
| Eglinton & Chung 2011 | UK | 44.6 (12.8) | 77 | | 100 | | CFS | progressive | 10 | NA | 5.58 | PDS | NA | 42 | 78 | 2 |
| Einsle et al. 2012 | Germany | 64.2 (7.9) | 28.3 | | NA | | coronary artery disease | progressive | NA | NA | 4 | SCID | NA | 1 | 60 | 2 |
| Einsle et al. 2012 | Germany | 50.6 (12.1) | 42.7 | | NA | | cancer | survivor | NA | NA | 2.7 | SCID | NA | 3 | 110 | 2 |
| Erickson & Steiner 2001 | USA | 20.4 (NA) | 65 | | 55 | | cancer | survivor | NA | 9.58 | 10.78 | SI-PTSD | 18 | 35 | 40 | 2 |
| Ford et al. 2015 | USA | 43.3 (11.0) | 52.1 | | 86.4 | | cancer | survivor | NA | 1 | 42.3 | IES | NA | 5 | 470 | 2 |
| Gao et al. 2015 | China | 56.2 (8.8) | 21.6 | | NA | | myocardial infarction | newly diagnosed | NA | NA | 0.02 | PCL-C | 38 | 25 | 97 | 2 |
| Goncalves et al. 2011 | UK | 61.0 (12.0) | 100 | | NA | | cancer | newly diagnosed, progressive | NA | NA | 0.5 | PDS | NA | 37 | 82 | 2 |
| Hahn et al. 2015 | USA | 51.0 (16.0) | 85 | | 74 | | cancer | survivor | NA | 40 | 11 | PCL-C | NA | 79 | 162 | 2 |
| Häuser et al. 2013 | Germany | 52.3 (8.8) | 93.9 | | NA | | FMS | newly diagnosed, progressive | NA | NA | 4.5 | PDS | NA | 179 | 395 | 3 |
| Häuser et al. 2015 | Germany | 51.0 (10.3) | 95.8 | | 89.25 | | FMS | newly diagnosed, progressive | 11.6 | NA | 9.6 | PDS | NA | 48 | 142 | 2 |
| Heim et al. 2009 | USA | 44.0 (NA) | 78.1 | | 71.7 | | CFS | progressive | NA | NA | NA | DTS | 40 | 22 | 112 | 2 |
| Ingerski et al. 2010 | USA | 13.2 (2.4) | 54.5 | | 15.4 | | HIV | progressive | 10.44 | 2.76 | 10.44 | UCLA PTSD RI | 38 | 1 | 12 | 2 |
| James et al. 2018 | USA | 62.2 (9.2) | 52 | | 40 | | diabetes mellitus type 2, cancer | newly diagnosed, progressive | NA | NA | 0.5 | IES | 26 | 17 | 52 | 2 |
| Kamibeppu et al. 2015 | Japan | 23.1 (5.0) | 58.3 | | NA | | cancer | survivor | NA | 8.3 | 14.8 | IES-R-J | 26 | 38 | 185 | 2 |
| Kim et al. 2020 | USA | 41.3 (11.5) | 58.97 | | NA | | migraine | progressive | 24.9 | NA | NA | PC-PTSD | 3 | 2 | 39 | 2 |
| Kornblith et al. 2010 | USA | 61.2 (11.0) | 100 | | 90.5 | | cancer | survivor | NA | 55.1 | 6.1 | PCL-C | NA | 0 | 42 | 2 |
| Landolt et al. 2012 | Switzerland | 10.7(2.5) | 30 | | NA | | diabetes mellitus type 1 | newly diagnosed | NA | NA | 1 | UCLA PTSD | 24 | 1 | 72 | 3 |
| Landolt et al. 2012 | Switzerland | 11.0 (2.7) | 32 | | NA | | cancer | newly diagnosed | NA | NA | 1 | UCLA PTSD | 24 | 3 | 77 | 3 |
| Langeveld et al. 2004 | the Netherlands | 24.0 (5.1) | 47 | | NA | | cancer | survivor | NA | 8 | NA | IES | NA | 199 | 500 | 2 |
| Lee & Santacroce 2007 | USA+Taiwan | 27.4 (5.5) | 62 | | 89 | | cancer | survivor | 2.0 | 11.4 | NA | PTSDI | 32 | 6 | 45 | 2 |
| Liang et al. 2019 | USA | NA | 47 | | NA | | hematopoietic stem cell transplantation (cancer) | survivor | NA | NA | 10.1 | PCL-5 | NA | 23 | 691 | 2 |
| Lin et al. 2020 | Taiwan | NA | 33 | | NA | | cancer (surgery) | survivor | NA | 58.7 | 4.2 | C-DTS | 44 | 21 | 188 | 1 |
| Mehnert & Koch 2007 | Germany | 54.9 (10.7) | 100 | | NA | | cancer | newly diagnosed, progressive | NA | NA | 0.04 | SCID | NA | 6 | 127 | 2 |
| Merriman et al. 2007 | UK | 73.8 (9.5) | 44.1 | | 99.01 | | stroke | survivor | NA | NA | 0.34 | PDS | NA | 31 | 101 | 2 |
| Muller et al. 2016 | France | NA | NA | | NA | | myocardial infarction | survivor | NA | NA | 2.67 | IES | NA | 2 | 41 | 1 |
| Nater et al. 2011 | USA | 50.6 (NA) | 83.72 | | 93.02 | | CFS | progressive | NA | NA | NA | DIS | NA | 4 | 43 | 2 |
| Oxlad & Wade 2006 | Australia | 63.3 (10.2) | 16 | | 95.8 | | ischaemic heart disease | progressive | NA | NA | NA | PDS | 23 | 9 | 119 | 2 |
| Palgi et al. 2011 | Israel | 57.3 (12.7) | 43.1 | | NA | | cancer | progressive | 1.11 | NA | 1.11 | PCL-C | 44 | 34 | 123 | 2 |
| Peltzer 2016 | South Africa | NA | 28.1 | | NA | | tuberculosis | newly diagnosed, progressive | NA | NA | 0.5 | PC-PTSD | 2 | 67 | 710 | 1 |
| Peterlin et al. 2009 | USA | 42.8 (12.4) | 90.6 | | 81.3 | | episodic migraine | progressive | NA | NA | NA | PCL-C | 44 | 89 | 398 | 2 |
| Radat et al. 2013 | France | 59.5 (13.8) | 52.2 | | NA | | polyneuropathy | progressive | NA | NA | NA | MINI | NA | 6 | 182 | 2 |
| Reme et al. 2011 | Norway | 45 (9.8) | 50.5 | | NA | | CLBP | progressive | 11 | NA | NA | MINI | NA | 3 | 565 | 2 |
| Robitaille 2019 | Canada | 27.3 (6.7) | 0 | | NA | | cancer | newly diagnosed | NA | 27.3 | 1 | PCL-C | 40 | 10 | 58 | 2 |
| Ross et al. 2019 | USA | 21.5 (2.1) | 59.1 | | 95.5 | | cancer | survivor | 6.7 | 14.8 | 6.7 | UCLA PTSD | 20 | 15 | 44 | 2 |
| Rourke et al. 2007 | USA | 24.8 (4.5) | 53.8 | | 87.9 | | cancer | survivor | NA | 8.7 | 13.6 | SCID | NA | 26 | 182 | 2 |
| Rusiewicz et al. 2008 | USA | 41.7 (11.6) | 54.7 | | 78.4 | | hematopoietic stem cell transplantation (cancer) | survivor | NA | 38.1 | 3.4 | PCL-C | 50 | 26 | 236 | 2 |
| Rutovic et al. 2019 | Croatia | NA | 37.6 | | NA | | ischaemic stroke | survivor | NA | NA | 0.25 | PCL-S | 50 | 11 | 85 | 1 |
| Santacroce et al. 2010 | USA | 21.0 (3.7) | 52 | | 85 | | cancer | survivor | NA | NA | 11.2 | RI | 12 | 20 | 20 | 2 |
| Semiz et al. 2013 | Turkey | 20.4 (1.9) | 72.8 | | NA | | migraine | progressive | NA | 17.11 | NA | SCID | NA | 6 | 169 | 2 |
| Shaw et al. 2009 | England | NA | 40 | | NA | | COPD | progressive | NA | NA | 0.08 | IES | 30 | 9 | 40 | 2 |
| Shemesh et al. 2006 | USA | 58.0 (9.0) | 10 | | NA | | myocardial infarction | newly diagnosed | NA | 58 | 0.5 | IES | 17 | 14 | 65 | 2 |
| Şişmanlar et al. 2012 | Turkey | 13.7 (2.4) | 50 | | NA | | diabetes mellitus type 1 | progressive | 3.77 | NA | NA | CPTS-RI | 12 | 9 | 42 | 2 |
| Smith et al. 2014 | USA | 62.9 (13.4) | 51 | | 85 | | cancer | nearly diagnosed | NA | NA | 10.2 | PCL-C | NA | 142 | 886 | 2 |
| Smitherman & Kolivas 2013 | USA | 18.9 (1.3) | 80 | | 78 | | migraine | progressive | NA | NA | NA | PCL-C | NA | 77 | 300 | 2 |
| Stuber et al. 2010 | USA | 31.9 (7.6) | 52.3 | | 87.5 | | cancer | survivor | NA | 8.21 | NA | PDS | NA | 589 | 6542 | 3 |
| Thieme et al. 2004 | Germany | 48.2 (10.3) | 100 | | NA | | FMS | progressive | 9.29 | NA | NA | SCID | NA | 9 | 115 | 2 |
| Tjemsland et al. 2016 | Norway | 50.0 (NA) | 100 | | NA | | cancer | nearly diagnosed | NA | NA | 1 | IES | 19 | 13 | 106 | 2 |
| Tremolada et al. 2016 | Italy | 19.3 (3.0) | 44.8 | | NA | | cancer | survivor | NA | 8.0 | NA | PTSD symptom check-list | 6 | 46 | 223 | 2 |
| Varela et al. 2013 | USA | NA | 50.5 | | 98.5 | | cancer | survivor | NA | 25 | 16 | PDS | NA | 37 | 105 | 2 |
| Wang et al. 2011 | UK | 75.2 (10.3) | 52.6 | | 100 | | stroke | nearly diagnosed | NA | NA | 0.3 | PDS | NA | 18 | 78 | 2 |
|  |  |  |  | |  | |  |  |  |  |  |  |  |  |  |  |
| Wenninger et al. 2013 | Germany | 28.9 (7.0) | 50.6 | | 89.0 | | cancer | survivor | 2.0 | 9.0 | NA | PDS - short form | 9 | 23 | 164 | 1 |
| Wiedemar et al. 2008 | Switzerland | 54 (9) | 25 | | NA | | myocardial infarction | survivor | NA | NA | 0.3 | CAPS | NA | 15 | 32 | 2 |

**Abbreviations:** CD, chronic somatic disease; CAPS, Clinican-administered PTSD scale interview; CFS, chronic fatigue syndrome; CLBP, chronic low back pain; C-DTS, Davidson Trauma Scale (Chinese version); DIS, Diagnostic Interview Schedule; CPTS-RI, Child Posttraumatic stress reaction index; DTS, Davidson Trauma Scale; FMS, fibromyalgia syndrome; IES, Impact of Event Scale; IES-R, Impact of Event Scale – Revised; IES-R-J, Impact of Event Scale – Revised (Japanese version); MINI, Mini International Neuropsychiatric Interview; NA, not available; %, percentage; PC-PTSD, Post-traumatic Stress Disorder Checklist in Primary Care; PCL-5, PTSD Checklist for the DSM-5; PCL-C, PTSD Checklist – Civilian Version; PCL-S, PTSD Checklist – Specific; PDS, Post-traumatic Stress Diagnostic Scale; PSS, Post-traumatic Symptom Scale; PTSDI, Post-traumatic Stress Disorder Index; RI, Posttraumatic Stress Disorder Reaction index; RoB, Risk of Bias (1=high, 2=moderate, 3=low); SCID, Structured Clinical Interview for DSM; SD, standard deviation; SI-PTSD, Structured Clinical Interview for PTSD; UCLA PTSD RI, The University of California at Los Angeles Posttraumatic Stress Disorder Reaction Index; UK, United Kingdom.

^a^ Studies are ordered alphabetically by author and then by year of study.
