# Supplementary material for: Prevalence and incidence of post-traumatic stress disorder and symptoms in people with chronic somatic diseases: A systematic review and meta-analysis
Source: Front Psychiatry. 2023 Jan 18;14:1107144. doi: 10.3389/fpsyt.2023.1107144 (PMC9889922; doi:10.3389/fpsyt.2023.1107144)
Supplement: Supplementary file 1 [file Data_Sheet_1.ZIP › S5. Study characteristics 12-month prevalence.docx]

**Supplementary table S5. Selected characteristics of the studies of 12-month prevalence of PTSD^a^ (k=3)**

| **Article label** | **Country** | **Age, mean (SD)** | **Female, %** | **Caucasian, %** | **Name of CD** | **Stage of CD** | **Timepoint diagnosis CD, years** | **Time of PTSD assessment, years after diagnosis** | **PTSD instrument** | **PTSD cases** | **Total** | **RoB ranking** |
| --- | --- | --- | --- | --- | --- | --- | --- | --- | --- | --- | --- | --- |
| Greer et al. 2011 | USA | 62.93 (NA) | 62.14 | 86.05 | cancer | survivor | NA | 5 | CIDI | 18 | 225 | 2 |
| Peterlin et al. 2011 | USA | 38.9 (12.9) | 80.5 | 77.2 | episodic migraine | progressive | NA | NA | CIDI | 36 | 251 | 2 |
| Seitz et al. 2010 | Germany | 30.7 (5.9) | 56 | NA | cancer | survivor | 15.78 | 13.68 | DIA-X/M-CIDI | 25 | 437 | 2 |

**Abbreviations:** CD, chronic somatic disease; CIDI, World Health Organization’s Composite International Diagnostic Interview; DIA-X/M-CIDI,
Expert System for Diagnosing Mental Disorders – computer-assisted version; NA, not available; %, percentage; RoB, Risk of Bias (1=high, 2=moderate, 3=low); SD, standard deviation.

^a^ Studies are ordered alphabetically by author and then by year of study.
