# Supplementary material for: Prevalence and incidence of post-traumatic stress disorder and symptoms in people with chronic somatic diseases: A systematic review and meta-analysis
Source: Front Psychiatry. 2023 Jan 18;14:1107144. doi: 10.3389/fpsyt.2023.1107144 (PMC9889922; doi:10.3389/fpsyt.2023.1107144)
Supplement: Supplementary file 1 [file Data_Sheet_1.ZIP › S6. Study characteristics lifetime prevalence.docx]

**Supplementary table S6. Selected characteristics of the studies of lifetime prevalence of PTSD^a^ (k=6)**

| **Article label** | **Country** | **Setting** | **Age, mean (SD)** | **Female, %** | **Caucasian, %** | **Name of CD** | **Stage of CD** | **Duration of CD, years** | **Timepoint diagnosis CD, years** | **Time of assessment, years since diagnosis** | **PTSD instrument** | **PTSD cases** | **Total** | **RoB ranking** |
| --- | --- | --- | --- | --- | --- | --- | --- | --- | --- | --- | --- | --- | --- | --- |
| Alter et al. 1996 | USA | outpatients | 54.0 (11.0) | 100 | 100 | cancer | survivor | 5.4 | NA | 5.4 | SCID | 6 | 27 | 2 |
| Mehnert & Koch 2007 | Germany | inpatients | 54.9 (10.7) | 100 | NA | cancer | newly diagnosed, progressive | NA | NA | 0.04 | SCID | 11 | 127 | 2 |
| Nicolson et al. 2010 | USA | general population | 56.0 (8.5) | 100 | 90 | fibromyalgia + osteoarthritis | progressive | NA | NA | NA | semi-structured assessment for the Genetics of Alcoholism-II | 13 | 70 | 2 |
| Peterlin et al. 2011 | USA | general population | 38.9 (12.9) | 80.5 | 77.2 | episodic migraine | progressive | NA | NA | NA | CIDI | 54 | 251 | 3 |
| Radat et al. 2013 | France | inpatients, outpatients | 59.5 (13.8) | 52.2 | NA | polyneuropathy | progressive | NA | NA | NA | MINI | 12 | 182 | 2 |
| Semiz et al. 2013 | Turkey | general population | 20.4 (1.9) | 72.8 | NA | migraine | progressive | 3.3 | 17.11 | NA | SCID | 11 | 169 | 2 |

**Abbreviations:** CD, chronic somatic disease; CIDI, World Health Organization’s Composite International Diagnostic Interview; MINI, Mini International Neuropsychiatric Interview; NA, not available; RoB, %, percentage; %, percentage; Risk of Bias (1=high, 2=moderate, 3=low); SCID, Structured Clinical Interview for DSM; SD, standard deviation.

^a^ Studies are ordered alphabetically by author and then by year of study.
