# Supplementary material for: Prevalence and incidence of post-traumatic stress disorder and symptoms in people with chronic somatic diseases: A systematic review and meta-analysis
Source: Front Psychiatry. 2023 Jan 18;14:1107144. doi: 10.3389/fpsyt.2023.1107144 (PMC9889922; doi:10.3389/fpsyt.2023.1107144)
Supplement: Supplementary file 1 [file Data_Sheet_1.ZIP › S7. Full Citation Details of Included Studies.docx]

**Supplementary Appendix S7**

**References of studies included in the systematic review**

Allen J, Willard VW, Klosky JL, et al. Posttraumatic Stress-Related Psychological Functioning in Adult Survivors of Childhood Cancer. *J Cancer Surviv*. 2018;12(2):216-223. doi:10.1007/s11764-017-0660-x

Alter CL, Pelcovitz D, Axelrod A, et al. Identification of PTSD in Cancer Survivors. *Psychosomatics*. 1996;37(2):137-143. doi:10.1016/S0033-3182(96)71580-3

Arigo D, Juth V, Trief P, Wallston K, Ulbrecht J, Smyth JM. Unique relations between post-traumatic stress disorder symptoms and patient functioning in type 2 diabetes. *J Health Psychol*. 2020;25(5):652-664. doi:10.1177/1359105317727839

Barakat LP, Kazak AE, Meadows AT, Casey R, Meeske K, Stuber ML. Families Surviving Childhood Cancer: A Comparison of Posttraumatic Stress Symptoms with Families of Healthy Children. *J Pediatr Psychol*. 1997;22(6):843-859. doi:10.1093/jpepsy/22.6.843

Ben-Zur H, Cohen M, Gouzman J. Posttraumatic growth moderates the effects of posttraumatic stress symptoms on adjustment and positive affective reactions in digestive system cancer patients. *Psychol Health Med*. 2015;20(6):685-696. doi:10.1080/13548506.2014.969747

Bruggimann L, Annoni JM, Staub F, von Steinbüchel N, van der Linden M, Bogousslavsky J. Chronic posttraumatic stress symptoms after nonsevere stroke. *Neurology*. 2006;66(4):513-516. doi:10.1212/01.wnl.0000194210.98757.49

Carpenter KM, Fowler JM, Maxwell GL, Andersen BL. Direct and Buffering Effects of Social Support Among Gynecologic Cancer Survivors. *Ann Behav Med*. 2010;39(1):79-90. doi:10.1007/s12160-010-9160-1

Dahl AA, Østby-Deglum M, Oldenburg J, et al. Aspects of posttraumatic stress disorder in long-term testicular cancer survivors: cross-sectional and longitudinal findings. *J Cancer Surviv*. 2016;10(5):842-849. doi:10.1007/s11764-016-0529-4

De Leeuw R, Schmidt JE, Carlson CR. Traumatic Stressors and Post-Traumatic Stress Disorder Symptoms in Headache Patients. *Headache*. 2005;45(10):1365-1374. doi:10.1111/j.1526-4610.2005.00269.x

DeCarvalho LT. Important Missing Links in the Treatment of Chronic Low Back Pain Patients. *J Musculoskelet Pain*. 2010;18(1):11-22. doi:10.3109/10582450903495981

Eglinton R, Chung MC. The relationship between posttraumatic stress disorder, illness cognitions, defence styles, fatigue severity and psychological well-being in chronic fatigue syndrome. *Psychiatry Res*. 2011;188(2):245-252. doi:10.1016/j.psychres.2011.04.012

Einsle F, Kraft D, Köllner V. Post-traumatic stress disorder (PTSD) in cardiology and oncology - which diagnostic tools should be used? *J Psychosom Res*. 2012;72(6):434-438. doi:10.1016/j.jpsychores.2012.02.008

Erickson SJ, Steiner H. Trauma and Personality Correlates in Long Term Pediatric Cancer Survivors. *Child Psychiatry Hum Dev*. 2001;31(3):195-213. doi:10.1023/A:1026477321319

Ford JS, Chou JF, Sklar CA, et al. Psychosocial Outcomes in Adult Survivors of Retinoblastoma. *J Clin Oncol*. 2015;33(31):3608-3614. doi:10.1200/JCO.2014.60.5733

Gao W, Zhao J, Li Y, Cao FL. Post-traumatic stress disorder symptoms in first-time myocardial infarction patients: Roles of attachment and alexithymia. *J Adv Nurs*. 2015;71(11):2575-2584. doi:10.1111/jan.12726

Gonçalves V, Jayson G, Tarrier N. A longitudinal investigation of posttraumatic stress disorder in patients with ovarian cancer. *J Psychosom Res*. 2011;70(5):422-431. doi:10.1016/j.jpsychores.2010.09.017

Greer JA, Solis JM, Temel JS, et al. Anxiety Disorders in Long-Term Survivors of Adult Cancers. *Psychosomatics*. 2011;52(5):417-423. doi:10.1016/j.psym.2011.01.014

Hahn EE, Hays RD, Kahn KL, Litwin MS, Ganz PA. Post-traumatic stress symptoms in cancer survivors: relationship to the impact of cancer scale and other associated risk factors. *Psychooncology*. 2015;24(6):643-652. doi:10.1002/pon.3623

Häuser W, Galek A, Erbslöh-Möller B, et al. Posttraumatic stress disorder in fibromyalgia syndrome: prevalence, temporal relationship between posttraumatic stress and fibromyalgia symptoms, and impact on clinical outcome. *PAIN*. 2013;154(8):1216-1223. doi:10.1016/j.pain.2013.03.034

Häuser W, Hoffmann E-M, Wolfe F, et al. Self-reported childhood maltreatment, lifelong traumatic events and mental disorders in fibromyalgia syndrome: a comparison of US and German outpatients. *Clin Exp Rheumatol*. 2015;33(1 0 88):S86-S92. https://www.clinexprheumatol.org/abstract.asp?a=8894. Accessed July 19, 2021.

Heim C, Nater UM, Maloney E, Boneva R, Jones JF, Reeves WC. Childhood Trauma and Risk for Chronic Fatigue Syndrome: Association With Neuroendocrine Dysfunction. *Arch Gen Psychiatry*. 2009;66(1):72-80. doi:10.1001/archgenpsychiatry.2008.508

Ingerski LM, Shaw K, Gray WN, Janicke DM. A pilot study comparing traumatic stress symptoms by child and parent report across pediatric chronic illness groups. *J Dev Behav Pediatr*. 2010;31(9):713-719. doi:10.1097/DBP.0b013e3181f17c52

James J, Harris YT, Kronish IM, Wisnivesky JP, Lin JJ. Exploratory study of impact of cancer-related posttraumatic stress symptoms on diabetes self-management among cancer survivors. *Psychooncology*. 2018;27(2):648-653. doi:10.1002/pon.4568

Kamibeppu K, Murayama S, Ozono S, et al. Predictors of Posttraumatic Stress Symptoms Among Adolescent and Young Adult Survivors of Childhood Cancer: Importance of Monitoring Survivors' Experiences of Family Functioning. *J Fam Nurs*. 2015;21(4):529-550. doi:10.1177/1074840715606247

Kim S-K, Chong CD, Dumkrieger G, Ross K, Berisha V, Schwedt TJ. Clinical correlates of insomnia in patients with persistent post-traumatic headache compared with migraine. *J Headache Pain*. 2020;21(1):33. doi:10.1186/s10194-020-01103-8

Kornblith AB, Mirabeau-Beale K, Lee H, et al. Long-Term Adjustment of Survivors of Ovarian Cancer Treated for Advanced-Stage Disease. *J Psychosoc Oncol*. 2010;28(5):451-469. doi:10.1080/07347332.2010.498458

Landolt MA, Ystrom E, Sennhauser FH, Gnehm HE, Vollrath ME. The mutual prospective influence of child and parental post-traumatic stress symptoms in pediatric patients. *J Child Psychol Psychiatry*. 2012;53(7):767-774. doi:10.1111/j.1469-7610.2011.02520.x

Langeveld NE, Grootenhuis MA, Voûte PA, De Haan RJ. Posttraumatic Stress Symptoms in Adult Survivors of Childhood Cancer. *Pediatr Blood Cancer*. 2004;42(7):604-610. doi:10.1002/pbc.20024

Lee YL, Santacroce SJ. Posttraumatic stress in long-term young adult survivors of childhood cancer: A questionnaire survey. *Int J Nurs Stud*. 2007;44(8):1406-1417. doi:10.1016/j.ijnurstu.2006.07.002

Liang J, Lee SJ, Storer BE, et al. Rates and Risk Factors for Post-Traumatic Stress Disorder Symptomatology among Adult Hematopoietic Cell Transplant Recipients and Their Informal Caregivers. *Biol Blood Marrow Transplant*. 2019;25(1):145-150. doi:10.1016/j.bbmt.2018.08.002

Lin YH, Kao CC, Pan IJ, Liu YH. Lower urinary symptoms, resilience, and post-traumatic stress symptoms among rectal cancer patients after surgery. *Jpn J Nurs Sci*. 2020;17(3):1-9. doi:10.1111/jjns.12320

Mehnert A, Koch U. Prevalence of acute and post-traumatic stress disorder and comorbid mental disorders in breast cancer patients during primary cancer care: A prospective study. *Psychooncology*. 2007;16(3):181-188. doi:10.1002/pon.1057

Merriman C, Norman P, Barton J. Psychological correlates of PTSD symptoms following stroke. *Psychol Health Med*. 2007;12(5):592-602. doi:10.1080/13548500601162747

Muller G, Flecher E, Lebreton G, et al. The ENCOURAGE mortality risk score and analysis of long-term outcomes after VA-ECMO for acute myocardial infarction with cardiogenic shock. *Intensive Care Med*. 2016;42(3):370-378. doi:10.1007/s00134-016-4223-9

Nater UM, Maloney E, Heim C, Reeves WC. Cumulative life stress in chronic fatigue syndrome. *Psychiatry Res*. 2011;189(2):318-320. doi:10.1016/j.psychres.2011.07.015

Nicolson NA, Davis MC, Kruszewski D, Zautra AJ. Childhood Maltreatment and Diurnal Cortisol Patterns in Women With Chronic Pain. *Psychosom Med*. 2010;72(5):471-480. doi:10.1097/PSY.0b013e3181d9a104

Oxlad M, Wade TD. Application of a chronic illness model as a means of understanding pre-operative psychological adjustment in coronary artery bypass graft patients. *Br J Health Psychol*. 2006;11(3):401-419. doi:10.1348/135910705X37289

Palgi Y, Shrira A, Haber Y, et al. Comorbidity of posttraumatic stress symptoms and depressive symptoms among gastric cancer patients. *Eur J Oncol Nurs*. 2011;15(5):454-458. doi:10.1016/j.ejon.2010.11.011

Peltzer K. Decline of common mental disorders over time in public primary care tuberculosis patients in South Africa. *Int J Psychiatry Med*. 2016;51(3):236-245. doi:10.1177/0091217416651258

Peterlin BL, Rosso AL, Sheftell FD, Libon DJ, Mossey JM, Merikangas KR. Post-traumatic stress disorder, drug abuse and migraine: New findings from the National Comorbidity Survey Replication (NCS-R). *Cephalalgia*. 2011;31(2):235-244. doi:10.1177/0333102410378051

Peterlin BL, Tietjen GE, Brandes JL, et al. Posttraumatic Stress Disorder in Migraine. *Headache*. 2009;49(4):541-551. doi:10.1111/j.1526-4610.2009.01368.x

Radat F, Margot-Duclot A, Attal N. Psychiatric co-morbidities in patients with chronic peripheral neuropathic pain: A multicentre cohort study. *Eur J Pain*. 2013;17(10):1547-1557. doi:10.1002/j.1532-2149.2013.00334.x

Reme SE, Tangen T, Moe T, Eriksen HR. Prevalence of psychiatric disorders in sick listed chronic low back pain patients. *Eur J Pain*. 2011;15(10):1075-1080. doi:10.1016/j.ejpain.2011.04.012

Robitaille R. *Symptoms of posttraumatic stress disorder in young males diagnosed with testicular or lymphatic cancer* [dissertation]. Montreal, CA: Université de Montréal; 2008.

Ross WL, Mitchell HR, Iyer NS, Santacroce SJ, Kadan-Lottick NS. Impact of Survivorship Care on Young Adult Survivors of Childhood Cancer With Post-Traumatic Stress Symptoms. *Oncol Nurs Forum*. 2019;46(1):33-43. doi:10.1188/19.ONF.33-43

Rourke MT, Hobbie WL, Schwartz L, Kazak AE. Posttraumatic Stress Disorder (PTSD) in Young Adult Survivors of Childhood Cancer. *Pediatr Blood Cancer*. 2007;49(2):177-182. doi:10.1002/pbc.20942

Rusiewicz A, DuHamel KN, Burkhalter J, et al. Psychological distress in long-term survivors of hematopoietic stem cell transplantation. *Psychooncology*. 2008;17(4):329-337. doi:10.1002/pon.1221

Rutovic S, Kadojic D, Dikanovic M, Solic K, Malojcic B. Prevalence and correlates of post-traumatic stress disorder after ischaemic stroke. *Acta Neurol Belg*. 2021;121(2):437-442. doi:10.1007/s13760-019-01200-9

Santacroce SJ, Asmus K, Kadan-Lottick N, Grey M. Feasibility and Preliminary Outcomes From a Pilot Study of Coping Skills Training for Adolescent-Young Adult Survivors of Childhood Cancer and Their Parents. *J Pediatr Oncol Nurs*. 2010;27(1):10-20. doi:10.1177/1043454209340325

Seitz DCM, Besier T, Debatin KM, et al. Posttraumatic stress, depression and anxiety among adult long-term survivors of cancer in adolescence. *Eur J Cancer*. 2010;46(9):1596-1606. doi:10.1016/j.ejca.2010.03.001

Semiz M, Şentürk IA, Balaban H, Yağız AK, Kavakçı Ö. Prevalence of migraine and co-morbid psychiatric disorders among students of Cumhuriyet University. *J Headache Pain*. 2013;14(1):1-6. doi:10.1186/1129-2377-14-34

Shaw RJ, Harvey JE, Bernard R, Gunary R, Tiley M, Steiner H. Comparison of Short-Term Psychological Outcomes of Respiratory Failure Treated by Either Invasive or Non-Invasive Ventilation. *Psychosomatics*. 2009;50(6):586-591. doi:10.1016/s0033-3182(09)70860-6

Shemesh E, Koren-Michowitz M, Yehuda R, et al. Symptoms of Posttraumatic Stress Disorder in Patients Who Have Had a Myocardial Infarction. *Psychosomatics*. 2006;47(3):231-239. doi:10.1176/appi.psy.47.3.231

Şişmanlar ŞG, Demirbaş-Çakir E, Karakaya I, et al. Posttraumatic stress symptoms in children diagnosed with type 1 diabetes. *Ital J Pediatr*. 2012;38(1):13. doi:10.1186/1824-7288-38-13

Smith SK, Samsa G, Ganz PA, Zimmerman S. Is there a relationship between posttraumatic stress and growth after a lymphoma diagnosis? *Psychooncology*. 2014;23(3):315-321. doi:10.1002/pon.3419

Smitherman TA, Kolivas ED. Trauma Exposure versus Posttraumatic Stress Disorder: Relative Associations With Migraine. *Headache*. 2013;53(5):775-786. doi:10.1111/head.12063

Stuber ML, Meeske KA, Krull KR, et al. Prevalence and Predictors of Posttraumatic Stress Disorder in Adult Survivors of Childhood Cancer. *Pediatrics*. 2010;125(5):e1124-e1134. doi:10.1542/peds.2009-2308

Thieme K, Turk DC, Flor H. Comorbid Depression and Anxiety in Fibromyalgia Syndrome: Relationship to Somatic and Psychosocial variables. *Psychosom Med*. 2004;66(6):837-844. doi:10.1097/01.psy.0000146329.63158.40

Tjemsland L, Søreide JA, Malt UF. Posttraumatic distress symptoms in operable breast cancer III: Status one year after surgery. *Breast Cancer Res Treat*. 1998;47(2):141-151. doi:10.1023/A:1005957302990

Tremolada M, Bonichini S, Basso G, Pillon M. Post-traumatic Stress Symptoms and Post-traumatic Growth in 223 Childhood Cancer Survivors: Predictive Risk Factors. *Front Psychol*. 2016;7(287):1-11. doi:10.3389/fpsyg.2016.00287

Varela VS, Ng A, Mauch P, Recklitis CJ. Posttraumatic stress disorder (PTSD) in survivors of Hodgkin's lymphoma: prevalence of PTSD and partial PTSD compared with sibling controls. *Psychooncology*. 2013;22(2):434-440. doi:10.1002/pon.2109

Wang X, Chung MC, Hyland ME, Bahkeit M. Posttraumatic stress disorder and psychiatric co-morbidity following stroke: The role of alexithymia. *Psychiatry Res*. 2011;188(1):51-57. doi:10.1016/j.psychres.2010.10.002

Wenninger K, Helmes A, Bengel J, Lauten M, Völkel S, Niemeyer CM. Coping in long-term survivors of childhood cancer: relations to psychological distress. *Psychooncology*. 2013;22(4):854-861. doi:10.1002/pon.3073

Wiedemar L, Schmid JP, Müller J, et al. Prevalence and predictors of posttraumatic stress disorder in patients with acute myocardial infarction. *Heart Lung*. 2008;37(2):113-121. doi:10.1016/j.hrtlng.2007.03.005
