# Supplementary material for: Prevalence and incidence of post-traumatic stress disorder and symptoms in people with chronic somatic diseases: A systematic review and meta-analysis
Source: Front Psychiatry. 2023 Jan 18;14:1107144. doi: 10.3389/fpsyt.2023.1107144 (PMC9889922; doi:10.3389/fpsyt.2023.1107144)

**Supplementary figure S9. Sensitivity analysis (leave one out) of point prevalence of PTSS in people with CD.**


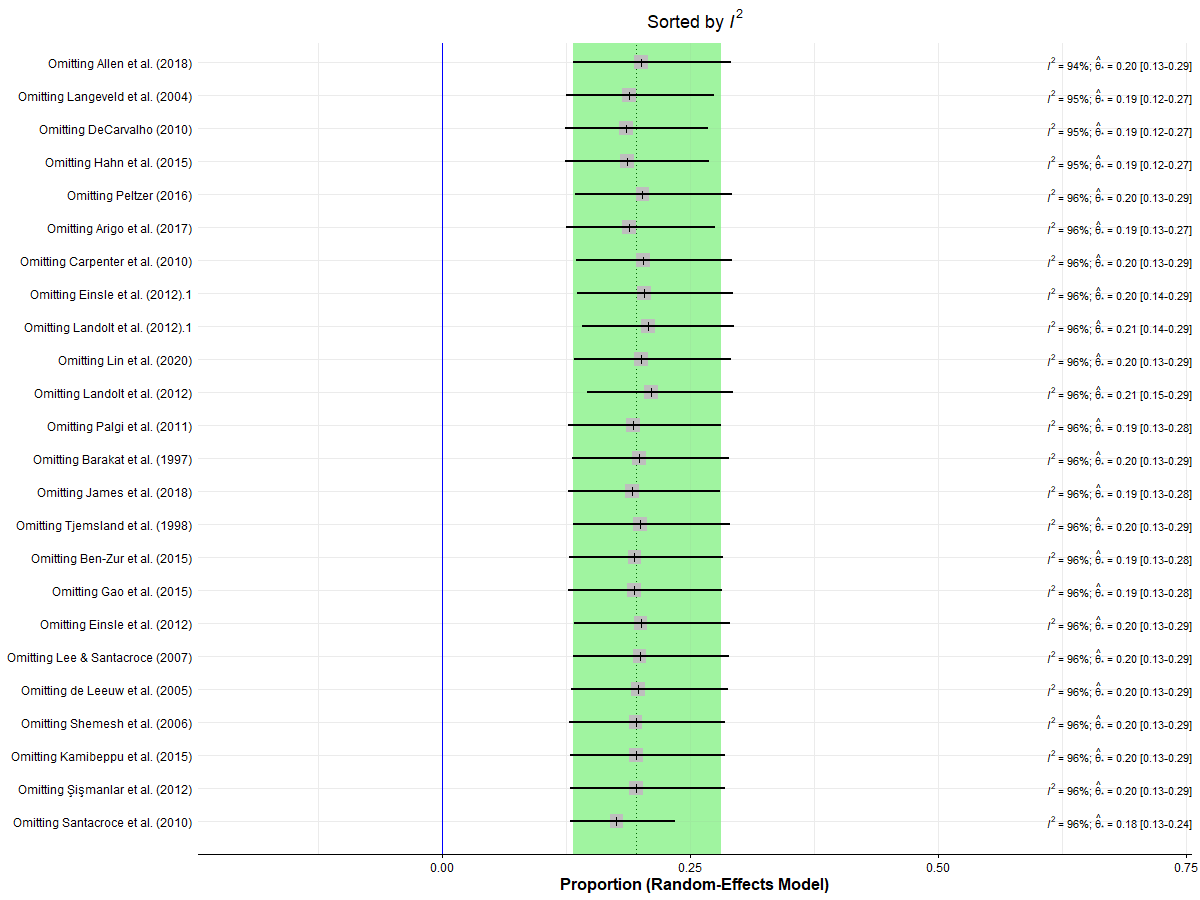

Supplement: Supplementary file 1 [file Data_Sheet_1.ZIP › S9. Sensitivity Analysis of PTSS Point Prevalence.docx]
